# Transcriptomics- and metabolomics-based integration analyses revealed the potential pharmacological effects and functional pattern of in vivo Radix Paeoniae Alba administration

**DOI:** 10.1186/s13020-020-00330-0

**Published:** 2020-05-24

**Authors:** Sining Wang, Huihua Chen, Yufan Zheng, Zhenyu Li, Baiping Cui, Pei Zhao, Jiali Zheng, Rong Lu, Ning Sun

**Affiliations:** 1 Department of Pathology, School of Basic Medical Sciences, Shanghai University of Traditional Chinese Medicine, 1200 CaiLun Ave, Pudong, 201203 Shanghai China; 2 Department of Physiology and Pathophysiology, School of Basic Medical Sciences, Fudan University, 130 DongAn Ave, Xuhui, 200032 Shanghai China; 3 College of Information and Computer Engineering, Northeast Forestry University, Harbin, China; 4 Public Laboratory Platform, School of Basic Medical Science, Shanghai University of Traditional Chinese Medicine, Shanghai, China

**Keywords:** Bioinformatics, Transcriptomics, Metabolomics, Radix Paeoniae Alba, Traditional Chinese medicine

## Abstract

**Background:**

Radix Paeoniae Alba (RPA) and other natural medicines have remarkable curative effects and are widely used in traditional Chinese Medicine (TCM). However, due to their multi-component and multi-target characteristics, it is difficult to study the detailed pharmacological mechanisms for those natural medicines in vivo. Therefore, their real effects on organisms is still uncertain.

**Methods:**

RPA was selected as research object, the present study was designed to study the complex mechanisms of RPA in vivo by integrating and interpreting the transcriptomic based RNA-seq and metabolomic based NMR spectrum after RPA administration in mice. A variety of dimension-reduction algorithms and classifier models were applied to the processing of high-throughput data.

**Results:**

Among serum metabolites, the contents of PC and glucose were significantly increased, while the contents of various amino acids, lipids and their metabolites were significantly decreased in mice after RPA administration. Based on the Gene Ontology (GO) and Kyoto Encyclopedia of Genes and Genomes (KEGG) databases, differential analysis showed that the liver was the site where RPA exerted a significant effect, which confirmed the rationality of “meridian tropism” in the theory in TCM. In addition, RPA played a role in lipid metabolism by regulating genes encoding enzymes of the glycerolipid metabolism pathway, such as 1-acyl-sn-glycerol-3-phosphate acyltransferase (Agpat), phosphatidate phosphatase (Lpin), phospholipid phosphatase (Plpp) and endothelial lipase (Lipg). We also found that RPA regulates several substance addiction pathways in the brain, such as the cocaine addiction pathway, and the related targets were predicted based on the sequencing data from pathological model in the GEO database. The overall effective pattern of RPA was intuitively presented with a multidimensional radar map through a self-designed model which found that liver and brain were mainly regulated by RPA compared with the traditional meridian tropism theory.

**Conclusions:**

Overall this study expanded the potential application of RPA and provided possible targets and directions for further mechanism study, meanwhile, it also established a multi-dimensional evaluation model to represent the overall effective pattern of TCM for the first time. In the future, such study based on the high-throughput data sets can be used to interpret the theory of TCM and to provide a valuable research model and clinical medication reference for the TCM researchers and doctors.

## Background

Radix Paeoniae Alba (RPA) is the dried root of the Chinese herbaceous peony buttercup plant, which is widely used in the treatment of liver diseases and emotional-related diseases in traditional Chinese medicine (TCM). In TCM theory, RPA is thought to have an effect of “nourishing blood, regulating menstruation, retaining “Yin”, stopping sweat, smoothing liver and relieving pain” according to *Pharmacopoeia of the People’s Republic of China* (Commission, 2015). According to modern pharmacological studies, paeoniflorin (PF), the main active ingredient in RPA, plays a role in the nervous and immune systems. PF significantly attenuated inflammatory pain by protecting neural progenitor cells and PC12 cells from oxidative stress damage through the ROS/PKC δ/NF-κB pathway and the PI3K/Akt-1 pathway [1–3]. PF also decreased caspase–3 activity and downregulated p–p38 MAPK expression in Alzheimer’s disease (AD) mice [4]. The anti-inflammatory effect also allowed PF to reduce cerebral infarct and neurological deficits in rats with ischemia–reperfusion injury, suggesting that PF might be used for treatment of stroke [5]. In addition, PF inhibited the activities and protein expression levels of inducible nitric oxide synthase, diminished IL-8 production, and thus exerted cardioprotective and hepatoprotective effects [6, 7].

Previous studies of RPA always use monomer components such as PF as the main research subject. However, changes in the organism caused by the whole herb itself are often different from those caused by a single component within the herb. Therapeutic efficacy of RPA has been confirmed by various clinical trials. However, due to the complex composition of RPA and the limitation of research techniques, the pattern of their complex effects and the microscopic changes have not been well interpreted. An unexpected and unbiased method to analyze the effects of natural medicines based on high-throughput data generated by different types of omics combinations is needed. After the development of “omics” and relevant technologies, their systematic strategies were highly consistent with the “holistic view” in the theory system of TCM and were gradually accepted by researchers. In addition, network pharmacology can clarify the synergistic effect of multicomponent- multitarget drugs, so it is also a suitable method to evaluate the efficacy and reveal the functional mechanisms of natural drugs [8].

Transcriptomics is one of the earliest omics technologies, which analyzes the changes in gene transcription caused by environmental or drug stimuli on an overall level. Metabolomics can evaluate the organisms’ response to conditional disturbances and yield biomarkers by identifying endogenous molecular metabolites that are quantitatively changed. Combined application of transcriptomics and metabolomics can systematically depict the complex relationship between the phenotypes and mechanisms. Although the mapping relationship between the metabolome and transcriptome is not direct in the information transmission sequence of the central principle [9], with increasing examples of their combined application, the analytical methods have become increasingly mature and reasonable. The integration analysis method based on prior knowledge can intuitively generate valuable insights [10], while the integration method based on metabolism-transcription pathways via KEGG and other public database can reveal the functional relationship among the targets more conveniently [11]. In addition, parametric models can also be constructed based on rate distortion criteria [12] or weighted gene correlation network analysis (WGCNA) [13]. These methods can overcome the limitations of established information and make the integration of omics data more efficient.

In this study, our aim of this study was to clarify the effect of RPA as a whole medical entity, not its single components, on live organisms to further compare with and analyze the belonging of the meridian theory in TCMs. Through the design of an in-laboratory model, a method that could present the action pattern of traditional Chinese natural medicines was established, which aimed to interpret the theory of TCM by scientific paradigm, and to provide a valuable research model and clinical medication reference for the TCM researchers and doctors. In addition, this method was more aligned than previous studies with the development trend of modern biomedicine towards systematization, which expanded the content of TCM theory and provided a new perspective for future relevant research.

## Methods and materials

### Quantitative high-performance liquid chromatography (HPLC) analysis of RPA

The contents of RPA in the sample preparations (180103, Kangqiao Traditional Chinese Medicine Co., Ltd., Shanghai) were determined by HPLC. The standard substance was PF (YZ-110736, National Institute for the Control of Pharmaceutical and Biological Products, Beijing). The chromatographic column was a QS-C18 Plus (4.6 mm × 50 mm, Puning Analysis Technology Co., Ltd., Shanghai). The mobile phase was acetonitrile-0.1% H_3_PO_4_ in gradient mode at a flow rate of 1.0 mL min^−1^, with a split ratio of 14:86, and the observation wavelength was 230 nm.

### Animals

Our research involved the utilization of laboratory animals under the supervision of the Fudan University Institutional Animal Care and Use Committee. The animals used were specific pathogen-free male C57BL/6 J mice (Slake Laboratory Animal Co. Ltd., Shanghai), which were bred for up to 8 weeks (weighted 20 ± 1.5 g) to adapt to the environment before the experiment. All animals were maintained in a room with regulated temperature (20 ± 2 °C) and relative humidity (40–70%). An artificial 12/12-h light/dark cycle was maintained, with lights turned on at 08:00 a.m.

### Preparation of RPA extract

RPA decoction pieces were soaked for 30 min at 50 °C with 6 times the volume of water, using a condensation reflux device to heat the mixture twice, each time for 1 h. The obtained decoction was made into a freeze-dried powder, and the powder extraction rate was calculated, which was 17.68% in this study. The dosage of RPA was calculated by the Meeh-Rubner formula coefficient k according to the common human adult clinical dose. The average dosage of administration was 3.9 crude drug (g)/weight (kg)/day.

### Transcriptome sequencing (RNA-seq)

Experimental mice were randomly divided into the control group and the RPA group (n = 3). After 7 days of intragastric administration, the vital organs (heart, liver, spleen, lung, kidney, brain and adrenal glands) were harvested for total RNA extraction. Transcriptome libraries were prepared by NGS Multiplex Oligos for Illumina (ExCell Biotech Co., Ltd.) according to the manufacturer’s instructions. After the libraries amplified, quality controlled, and then run on an Illumina HiSeqX10 platform with a paired-end 150 bp sequencing strategy. Fragments per kilobase of exon per million mapped reads (FPKM) were used to compare gene expression differences between different samples and the software OmicsBean (http://www.omicsbean.com:88/) was used to identify and analyze the differentially expressed genes (DEGs), with fold change ˃  1.5 and FDR ˂ 0.05 used as the criteria for significant differences between the two groups as described in previous studies [14].

### Untargeted metabolomics analysis (NMR)

Mice were grouped and treated in the same way as those for the transcriptome analysis (n = 7). At the end of the experiment, all animals were fasted except water for 12 h and sacrificed following isoflurane anesthesia. Serum samples were obtained for each group using a standard protocol (removed the hemolytic sample). Each serum sample (30 μL) was mixed with 30 μL of phosphate buffer (45 mM, pH 7.43) and then transferred into NMR tube and used directly for detection. The analysis methods of NMR spectroscopy referred to a previously published paper [15]. Data analysis was performed with the software package SIMCA-P + (V14.0, Umetrics, Sweden) and a MATLAB script (MATLAB V7.1, Mathworks Inc., USA). Feature extraction data analysis and OPLS-DA were carried out. All models were further tested with a T-test for significance of intergroup differentiations (with p < 0.05 as a significant level). MetaboAnalyst 4.0 (https://www.metaboanalyst.ca) was used for pathway enrichment analysis [16].

### Network pharmacology

The Traditional Chinese Medicine Systems Pharmacology Database and Analysis Platform (TCMSP http://lsp.nwu.edu.cn/index.ph) was used to select the active ingredients of RPA by combining the oral absorption (OB > 30%) and drug type index (DL > 0.18). Corresponding targets were also identified by searching the TCMSP. Targets’ symbol names were obtained through a Perl language script, which was built in-house UniProt database. The transformation between the target symbol and entrezID and the enrichment analysis was performed using R packages (“org.Hs.e.g.db” and “pathview”).

### A multidimensional algorithm model was established based on multiorgan transcriptomics data

PCA was used to reduce the dimension of the 7 transcriptomes datasets, and the first principal component (PC01) was selected as representative, of which the contribution rate was calculated. Next, the increased-magnitude was calculated in the RPA group relative to the CON group through the formula:

$$ \varvec{y}_{\varvec{i}} = \left( {\varvec{PC}01_{{\varvec{iarvP}}} - \varvec{PC}01_{{\varvec{iarvC}}} } \right)/\left( {\varvec{PC}01_{{\varvec{imax}}} - \varvec{PC}01_{{\varvec{imin}}} } \right) $$

where $y_{i}$ is the amplification of RPA group, $PC01_{iarvP}$ is the average $PC01$ of the i_st_ organ in the RPA group, $PC01_{iarvC}$ is the average $PC01$ of the i_st_ organ in the CON group, $PC01_{imax}$. is the maximum $PC01$ of the two groups, and $PC01_{imin}$ is the minimum $PC01$ of both.

Third step, according to the number of different organs genes,$y_{i}$ was given different weights. Fuzzy set theory was used to design the weight, four trapezoidal membership function and membership function was designed as following and shown in Additional file 1: Fig. S1, the four function to represent the “least”, “less”, “more” and “most” of four conditions which values were set to 0.3, 0.5, 0.7, 0.9, then the four membership degree values of DEGs in different organs were calculated. The function serial number corresponding to the maximum membership value was calculated by using the maximum operator rule and converted to the corresponding weight value. The weight values corresponding of seven transcriptome genes were shown in Additional file 2: Table S1.

$$ \varvec{\mu}_{1} \left( \varvec{x} \right) = \left\{ \begin{aligned} & 1 \quad \quad \quad \quad  \quad x < 300 \\ & \frac{{400 - \varvec{x}}}{100} \quad \quad \;300 \le x \le 400 \\ & 0\quad \quad \quad \quad \quad  x > 400 \\ \end{aligned} \right. $$

$$ \varvec{\mu}_{2} \left( \varvec{x} \right) = \left\{ \begin{aligned}   & 0\quad \quad \quad \quad x < 300 \\ & \frac{{\varvec{x} - 300}}{100}\quad \;300 \le x \le 400 \\ & 1\quad \quad \quad \quad 400 \le x \le 550  \\ & \frac{{650 - \varvec{x}}}{100}\quad \;550 \le x \le 650 \\ & 0\quad \quad \quad \quad x > 650 \\ \end{aligned} \right. $$

$$ \varvec{\mu}_{3} \left( \varvec{x} \right) = \left\{ \begin{aligned} & 0\quad \quad \quad \quad x < 550 \\ & \frac{{\varvec{x} - 550}}{100}\quad \;550 \le x \le 650 \\ & 1\quad \quad \quad \quad 650 \le x \le 800  \\ & \frac{{900 - \varvec{x}}}{100}\quad \;800 \le x \le 900 \\ & 0\quad \quad \quad \quad x > 900 \\ \end{aligned} \right. $$

$$ \varvec{\mu}_{4} \left( \varvec{x} \right) = \left\{ \begin{aligned} & 0\quad \quad \quad \quad x > 800 \\ & \frac{{\varvec{x} - 800}}{100}\quad \;800 \le x \le 900 \\ & 1\quad \quad \quad \quad x > 900 \\ \end{aligned} \right. $$

## Results

### RPA caused changes in serum metabolites in mice

**Fig. 1:**
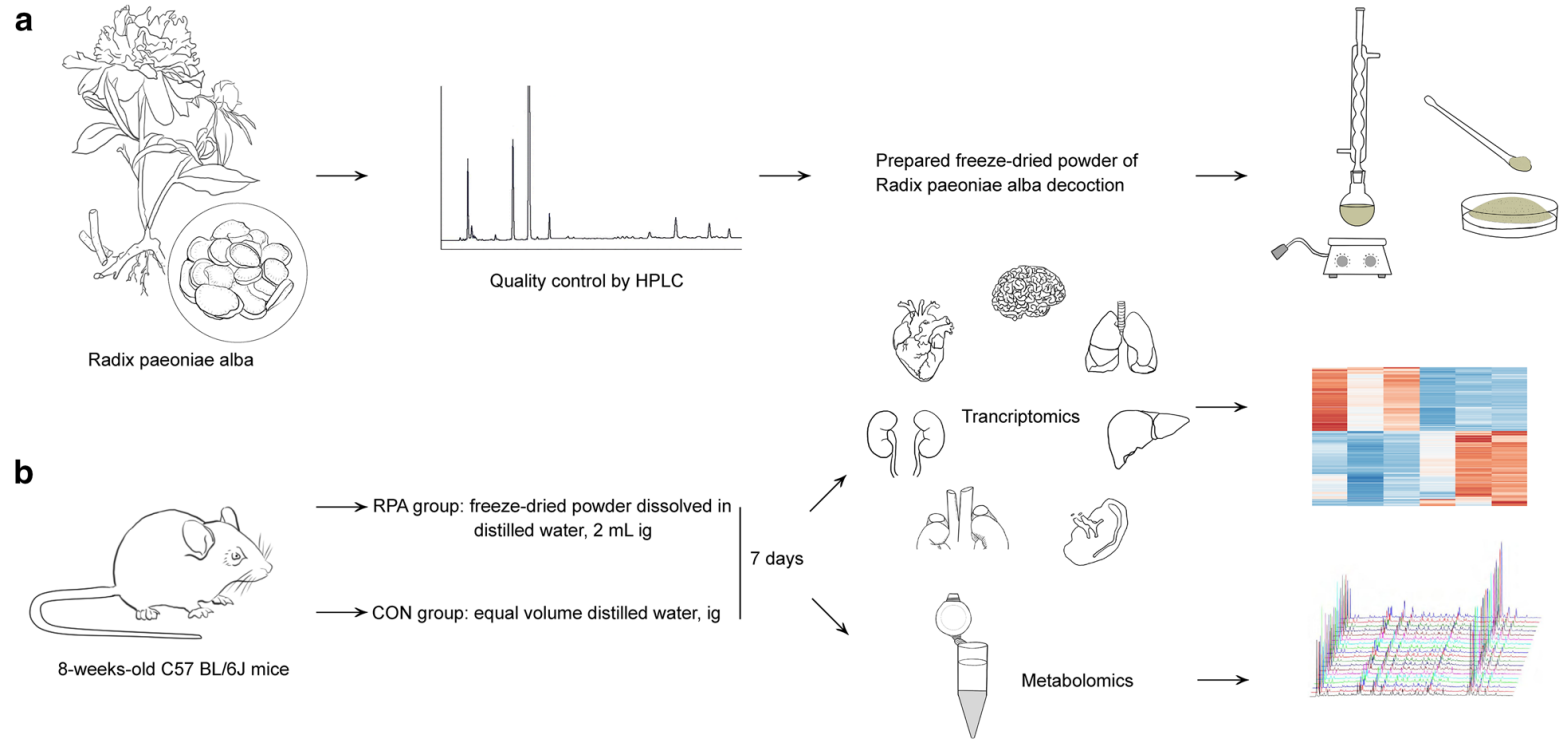
Overview of the experimental design. **a** Referring to *Pharmacopoeia of the People’s Republic of China* for quality control by HPLC, RPA was prepared as a freeze-dried powder for in vivo administration in mice. **b** Vital organs were harvested for transcriptomics analyses, and serum was collected for metabolomics analyses (n = 3 samples, intragastric administration of RPA for 1 week)

**Fig. 2:**
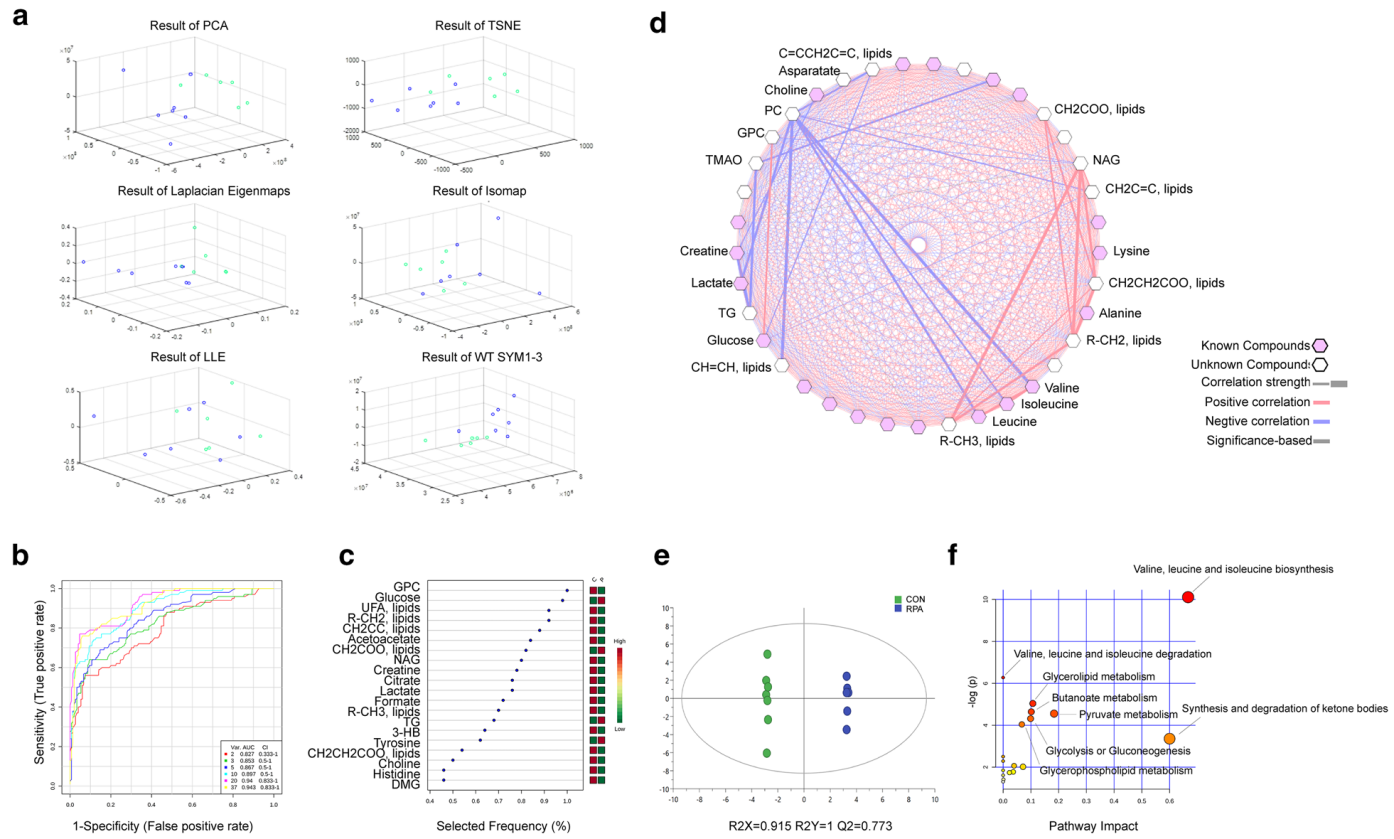
RPA administration induced changes in the serum metabolism profile in mice. **a** Feature extraction analyses based on six data dimension-reduction algorithms (n = 7 CON group and 6 RPA group samples). **b** ROC curves based on the CV performance by the SVM classifier, the default is the ROC curves from all models averaged from all CV runs, and the 95% confidence interval can be computed in this case. **c** Model of 20 features that were selected as screening criteria, AUC = 0.94. **d** Correlation analysis network among all identified serum metabolites. **e** Differential metabolite analysis model between groups based on OPLS-DA. **f** Pathways enriched by differential metabolites-based KEGG; the redder the color is, the more significant the result. p < 0.05. *PCA* principal component analysis, *t-SNE* T-distributed stochastic neighbor embedding, *ISOMAP* isometric mapping, *LLE* locally linear embedding, *WT* wavelet transform, *ROC* receiver operating characteristic, *CV* cross-validation, *AUC* area under the curve

To ensure the safety and effectiveness of RPA, quality control by HPLC was carried out for the drug used in the experiment, and the test results were in line with the descriptions in *Pharmacopoeia of the People’s Republic of China* [17]. Reports and typical fingerprints of standard and test samples are displayed in the Additional file 3: Fig. S2 and Additional file 4: Table S2. Next, the experiment was carried out according to the flow design shown in Fig. 1. After NMR serum metabolomics experiments, of which the representative spectra and primary signals are displayed in the Additional file 5: Fig. S3, feature extraction analyses of data were conducted to explore the rationality of the model through various dimension-reduction methods. The first three panels in Fig. 2A revealed a surface to completely distinguish the two groups of data after rotation. This finding suggested that the choice of dimension-reduction algorithm must be combined with a classifier. We therefore categorized the data by the Back-Propagation Neural Network (BPNN), Support Vector Machine (SVM), Random Forest (RF), Naive Bayesian (NB) and k-Nearest Neighbor (kNN) approaches at this stage (Additional file 6: Table S3). The discernment accuracies of the data were calculated based on dimension-reduction algorithm under different classification algorithms and again calculated on classification algorithms under different dimension-reduction algorithms (Tables 1, 2). It can be concluded from the results that the dimensionality reduction data obtained by the PCA algorithm could distinguish the samples of the control (CON) group and those of the RPA group most effectively. The average recognition time of a single sample with the SVM classification algorithm was far less than that with the other algorithms. Therefore, it was feasible and effective to use serum metabolites to distinguish the samples of the CON and RPA groups. If the number of samples increased gradually, the recognition accuracy could be further improved.

**Table 1:** The average recognition accuracy of each dimension reduction algorithm under different classification algorithms

	PCA	TSNE	LAP	ISO	LLE	WT
Mean recognition (%)	88	80	72	84	72	72

**Table 2:** The average recognition accuracy of each classification algorithm under different dimensionality reduction algorithms

	NB	RF	BPNN	kNN	SVM
Mean recognition (%)	86.7	76.7	53.3	86.7	86.7
Meantime (ms)	18.0016	310.1741	55.0986	49.5707	0.6827

Since SVM showed a good effect on the classification of small samples, we chose the SVM classifier to calculate the receiver operating characteristic (ROC) curve (Fig. 2b). Metabolites with high frequency in the model (Fig. 2c, 20 features, AUC = 0.94), such as glycerophosphocholine (GPC), glucose, acetoacetate, and a variety of unsaturated fatty acids, can be selected as serum biomarkers to show the effect of RPA. Afterward, the Metscape tool [18] was used to perform a correlation analysis, and the results indicated that phosphorylcholine (PC) and *N*-acetylated protein (NAG) played a significant role in the correlation network. Among them, PC had a significant negative correlation with various amino acids, including valine, isoleucine, leucine, and some lipids, while NAG had a significant positive correlation with various small molecular fatty acids in the network (Fig. 2d).

To identify those significantly different metabolites induced by RPA administration, OPLS-DA was conducted for the metabolomics data, and the model parameters were obtained as R2X = 0.915, R2Y = 1, and Q2 = 0.773, which indicated that the model was stable and reliable and had high predictive ability (Fig. 2e). Variable importance in projection (VIP) > 1.0 was used as the screening standard for differential metabolites, and 16 statistically significant differential metabolites were finally determined (Table 3), including mainly choline (PC and GPC), amino acids (leucine and valine), lipids [CH_2_C=C, C=CCH_2_C=C, R-CHI, R-CH_3_, CH_2_CH_2_COO, CH_2_COO and triglycerides (TGs)], ketones (acetoacetate, glucose, pyruvate and lactate) and glycoproteins (NAG), which were the representative significantly changed metabolites after RPA administration in mice. Among them, the contents of PC were significantly increased, while the contents of various lipids and their metabolites (acetoacetic acid) were significantly decreased, which may be due to the function of choline to regulate lipid metabolism [19]. In addition, amino acids were significantly reduced, whereas glucose was significantly increased. Metabolites such as pyruvate and lactic acid were also significantly reduced in mice after RPA administration. These results suggested that RPA participated in regulating glucose, amino acids, lipids, and other energy metabolism processes in the body. Based on the KEGG database, enrichment analysis was performed according to the differential metabolites to demonstrate the topological properties. We found that there were mainly 18 pathways involved (Additional file 7: Table S4), and the results were largely consistent with an analysis based on the Small Molecule Pathway Database (SMPDB) (Additional file 8: Fig. S4A). Integrating the p values between different pathways, the results showed that valine, leucine and isoleucine biosynthesis and degradation; butanoate metabolism; pyruvate metabolism; glycolysis or gluconeogenesis; glycerolipid metabolism; glycerophospholipid metabolism; and the synthesis and degradation of ketone bodies changed significantly (Fig. 2f). These results showed that RPA administration significantly affect the above metabolism pathways.

**Table 3:** List of significant differential metabolites from NMR

Metabolites	VIP score	*P* value
Glycerophosphocholine (GPC)	1.55808	0.000531147
CH2C=C, lipids	1.45282	0.002158383
Lactate	1.4504	0.002223364
Glucose	1.44983	0.00216794
*N*-Acetylated glycoproteins (NAG)	1.4238	0.002973999
phosphorylcholine (PC)	1.42161	0.00316847
R-CH_2_, lipids	1.39571	0.003988276
CH2CH2COO, lipids	1.32479	0.007758848
Acetoacetate	1.31558	0.008085364
R-CH3, lipids	1.3114	0.008461332
CH2COO, lipids	1.2774	0.011238583
C=CCH2C=C, lipids	1.27295	0.011567084
Triglycerides (TG)	1.13749	0.030249594
Leucine	1.1298	0.030957902
Pyruvate	1.06733	0.044451519
Valine	1.06733	0.044568734

### Liver is the major organ that affected by RPA administration

**Fig. 3:**
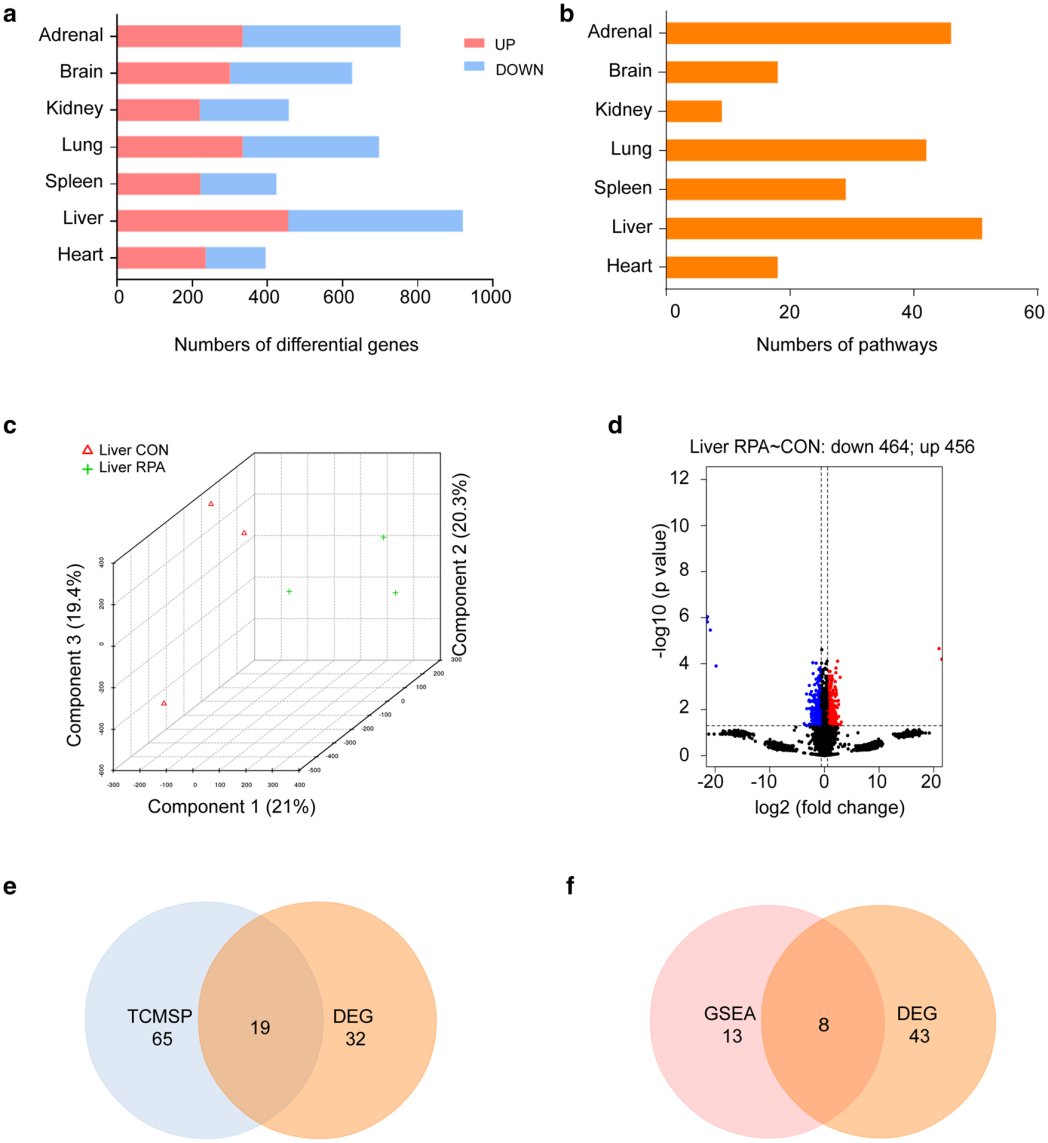
Integrated analyses of the liver revealed that it was the site where RPA exerted a major effect. **a** RPA induced a number of upregulated genes (red) and downregulated genes (blue) in various vital organs (p ˂ 0.05, cutoff = 1.5). **b** Number of pathways enriched with DEGs in each organ, based on KEGG. **c** Three-dimensional PCA revealed the overall intergroup separation of the liver transcriptomes. **d** Volcano plot showing the upregulated genes (red) and downregulated genes (blue) in liver tissue. **e** Venn diagram showing the overlapping pathways enriched with DEGs from our results and previously identified targets of RPA in the TCMSP. **f** Venn diagram showing the overlapping pathways enriched with DEGs by GSEA. Detailed result diagrams are shown in additional files

To study the regulation of gene expression by RPA, a comprehensive transcriptomics analysis was conducted on several vital organs, and the results showed that RPA could cause different degrees of changes in the transcripts of each organ (Fig. 3a). RPA is considered as a main medicine for regulating the liver meridian in TCM theory. Extensive RPA clinical application evidences in liver function regulation and liver diseases treatment were also constantly observed. Our transcriptomics analysis results indeed confirmed the traditional medical experiences. Compared with that of other organ tissues, the liver’s response to RPA administration was the most significant regardless of the number of DEGs or the significantly enriched pathways (Fig. 3b). The liver is also the main place of energy metabolism, which was reflected in the metabolomics results obtained in this study. We next systematically analyzed the liver transcriptome. The PCA result suggested that there was a good separation between the groups (Fig. 3c), with 456 genes were upregulated and 464 genes were downregulated (Fig. 3d). Next, pathway enrichment analysis was carried out for DEGs. According to the KEGG database, pathways were clustered into classes (subcategories) such as metabolism (carbohydrate metabolism, lipid metabolism, and nucleotide metabolism), environmental information processing (signal transduction and signaling molecules and interaction), cellular processes (transport and catabolism, cell growth and death, and cellular community), organismal systems (immune system, endocrine system, development, and environmental adaptation) and human diseases (cancer, neurodegenerative diseases, substance dependence, and infectious diseases: bacterial, viral and parasitic) (Additional file 9: Table S5).

RPA plays a series of roles in TCM theory, including collecting “yin” in liver, nourishing the liver blood, softening the liver body, and relieving acute liver diseases. However, these descriptions are often difficult to understand by modern medicine. Therefore, we conducted a network pharmacology analysis on RPA administration to interpret its in vivo efficacy. Thirteen main ingredients of RPA were obtained from the TCMSP (Additional file 10: Table S6), which involved 61 targets and 84 pathways enriched by the R package [20]. Nineteen pathways overlapped with the pathways of liver transcriptomics enrichment caused by RPA administration (Fig. 3e and Additional file 8: Fig. S4B), which could be considered the stable part of the pharmacological effects of RPA, as well as proving laterally the rationality of our study.

**Fig. 4:**
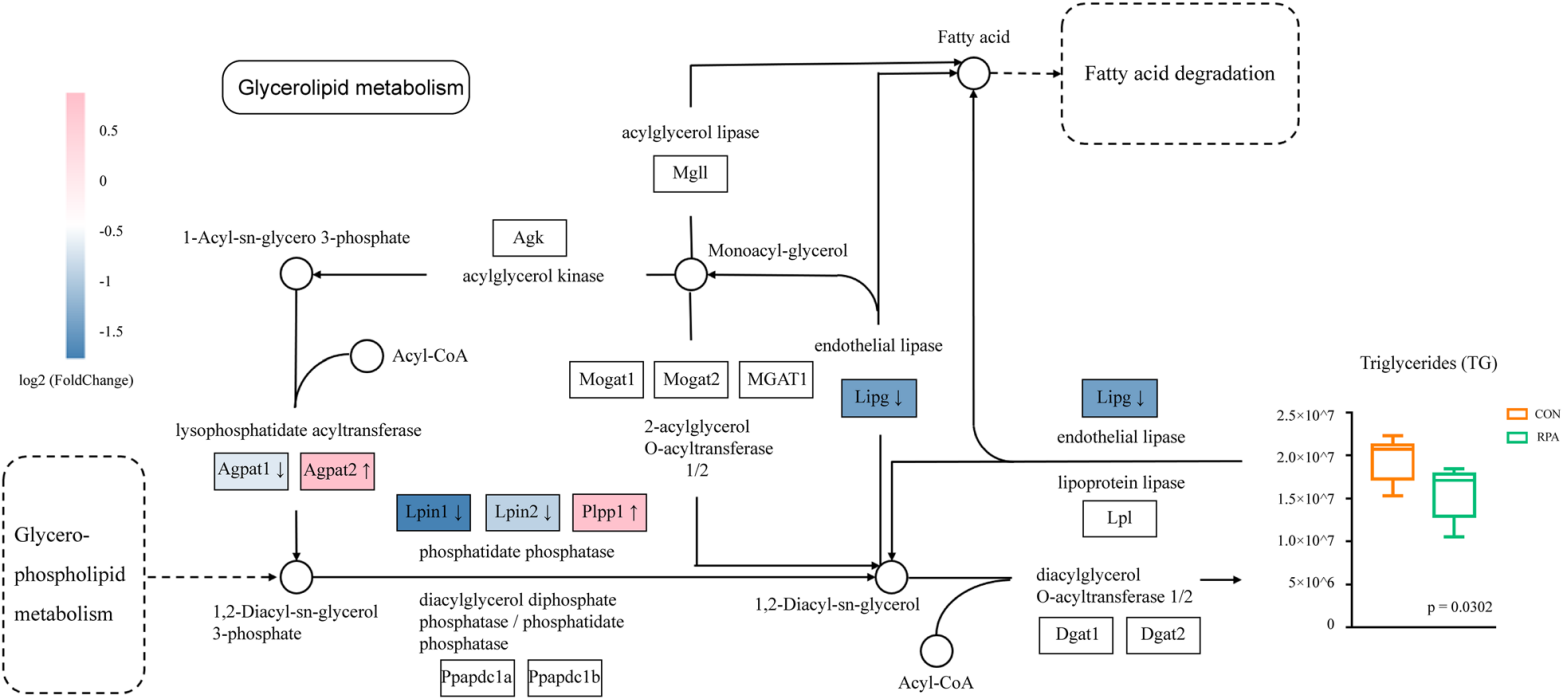
A Reconstructed pathway map of glycerolipid metabolism containing key genes and metabolites that were regulated. The level of TGs among serum metabolites decreased significantly which represented in the histogram. *DEGs* differentially expressed genes, *TG* triglyceride

Since the RPA induced pathway changes were most extensive in the liver, in order to observe the most representative pathways, Gene Set Enrichment Analysis (GSEA) was conducted on the liver transcriptome (Additional file 11: Table S7), and when the result of DEGs pathway enrichment overlapped with that of GSEA, 8 pathways, including leukocyte transendothelial migration, natural killer cell mediated cytotoxicity, hematopoietic cell lineage, leishmaniasis, prion diseases, cell adhesion molecules, lysosome and glycerolipid metabolism had significant differences and consistent changes under both analysis methods (Fig. 3f, Additional file 12: Fig. S5 and Additional file 13: Table S8). When combined with the metabolomics results, it was found that the glycerolipid metabolism pathway was enriched in both results (Additional file 8: Fig. S4C). In this pathway, several important genes, such as Agpat1, Agpat2, Lpin1, Lpin2, Plpp1 and Lipg, were regulated to varying extents, resulting in an overall decrease in lysophospholipid acyltransferase, phosphatidate phosphatase, and endothelial lipase. Subsequently, TGs and other fatty acids among serum metabolites were reduced, suggesting that RPA played an important regulatory role in lipid metabolism (Fig. 4 and Additional file 14: Fig. S6).

### RPA regulated the central nervous system

**Fig. 5:**
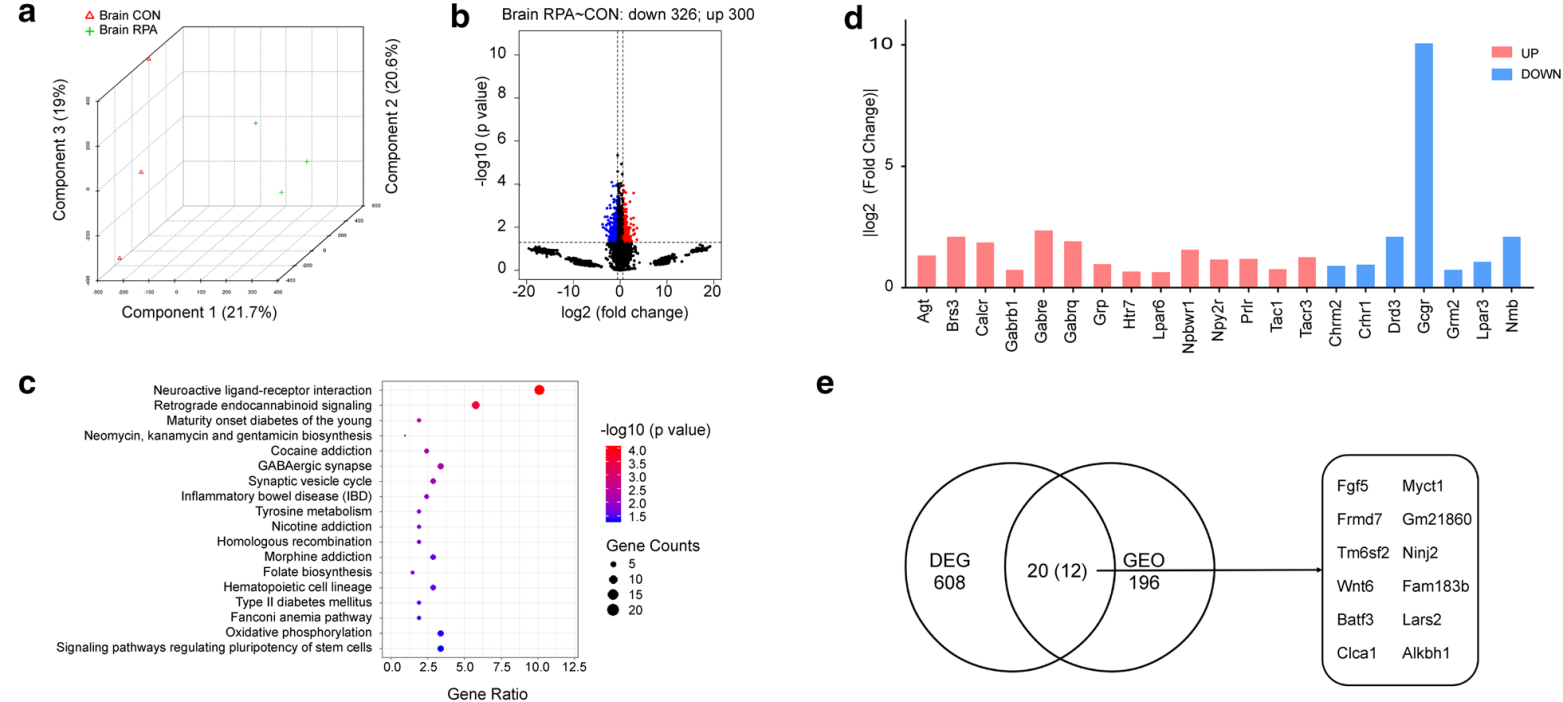
RPA had a significant effect on the transcriptome of brain tissue. **a** Three-dimensional PCA revealed the overall intergroup separation of the brain transcriptomes. **b** Volcano plot showing the upregulated genes (red) and downregulated genes (blue) in brain tissue. **c**KEGG enrichment bubble plot of DEGs in the brain transcriptomes (pathway p ˂ 0.05). **d** Target genes were significantly upregulated (red) and downregulated (blue) by RPA in the neuroactive ligand-receptor interaction pathway. **e** Venn diagram showing the potential targets of RPA on the cocaine addiction model based on RNA-seq data obtained from the GEO database

Among the differential metabolites after RPA administration, the contents of GPC and PC were simultaneously increased. Glycerophospholipid metabolism (the upstream pathway of glycerolipid metabolism, Fig. 4), in which PC and GPC were enriched, was also regulated in the liver transcriptomics results, further confirming the role of RPA in liver lipid metabolism. Interestingly, choline molecules are not only important components of lipid metabolism in the liver [21] but also key substances for brain function and information transmission [22]. In our metabolomics results, PC, the upstream substance of citicoline (by choline-phosphate cytidylyltransferase [23], was significantly elevated (Table 3). Of note, citicoline has been clinically used in the treatment of cerebral ischemic diseases such as stroke or vascular cognitive impairment [24–26]. PC is also a source of choline, which can be converted into acetylcholine by choline *O*-acetyltransferase and phosphocholine phosphatase [27]. We next therefore analyzed the brain transcriptome. We found PCA presented a similar form to the liver, suggesting that RPA administration could effectively separate the transcriptome data of the RPA group from that of the CON group (Fig. 5a). There were 300 upregulated genes and 326 downregulated genes (Fig. 5b) in the brain after RPA administration, which were enriched in 18 pathways (Fig. 5c). Among them, the most significantly changed pathway was the neuroactive ligand-receptor interaction pathway, which is related to a variety of nerve activities (Additional file 15: Fig. S7, mmu04080). Genes related to serotonin receptor, neuropeptide receptor, neuroregulatory peptide receptor, and nucleotide receptor in the pathway were upregulated, while genes related to acetylcholine receptor, dopamine receptor, and lysophosphatidic acid receptor were downregulated (Fig. 5d). Interestingly, in the enrichment results for disease class, various substance addiction pathways, such as those for morphine, nicotine, cocaine, etc., were found. Such pharmacological effects of RPA on substance addiction have never been reported previously (Additional file 16: Fig. S8). To explore the targets of RPA on substance addiction, the pathway of cocaine addiction was taken as a model and the transcriptome sequencing datasets (GSE108836) of the associated disease model were obtained by searching the Gene Expression Omnibus (GEO) database. A Venn analysis was conducted on the DEGs of the model group and our RPA group. Twelve DEGs (Myct1, Gm21860, Ninj2, Fam183b, Lars2, Alkbh1, Fgf5, Frmd7, Tm6sf2, Wnt6, Batf3, and Clca1) were found to be regulated inversely among the overlap genes, which may be possible targets of RPA to disrupt the cocaine addiction process (Fig. 5e).

### A multidimensional algorithm model of the overall effective pattern for RPA administration in vivo

**Fig. 6:**
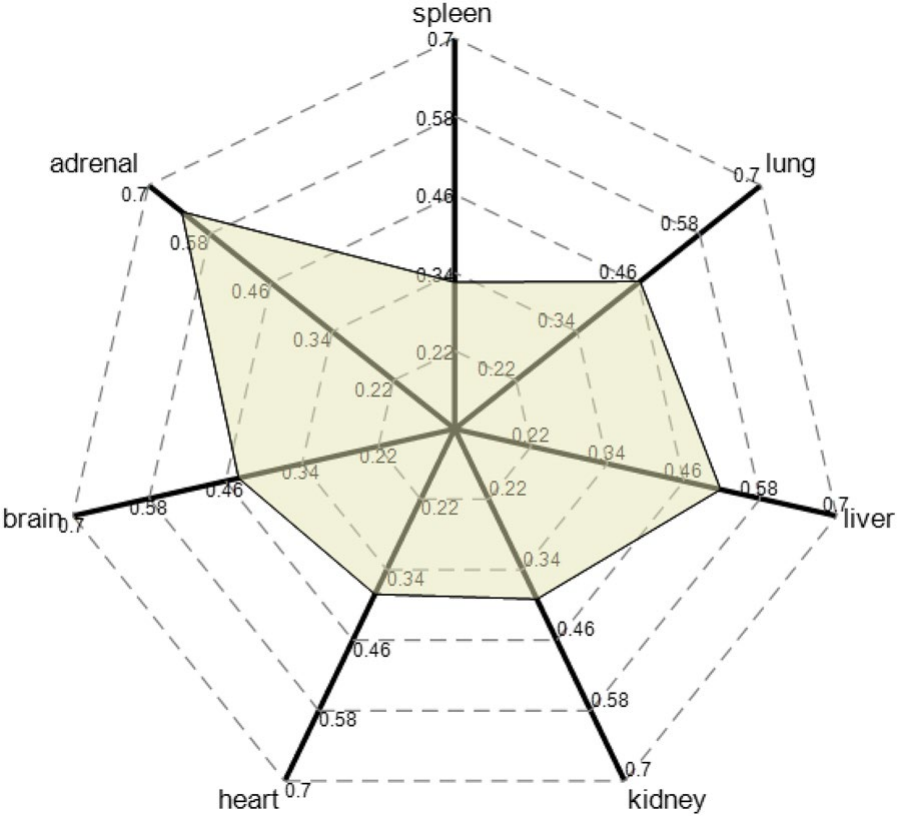
A multidimensional algorithm model was established based on the multiorgan transcriptomics data

To evaluate the effect pattern of RPA from the perspective of systemic changes caused by in vivo drug administration, a multidimensional algorithm model was established based on multiorgan transcriptomics data and applied to the analysis. The higher the contribution rate of PC01 was, the more representative it was among the entire transcriptome sample of PCA (Additional file 17: Table S9). The results showed that most contribution rates of PCs were more than 70% and the highest of them was more than 95%, indicating that the PC01 of transcriptomes can effectively represent the whole sample. The results of each organization can be obtained by multiplying $y_{i}$ and the corresponding weight value. Finally, 7 values obtained were drawn into a multidimensional radar map, which can intuitively present the overall effective pattern of RPA administration in vivo (Fig. 6). As previously mentioned, the liver and brain are the main sites where RPA exerted an effect. In addition, the intensity of the adrenal transcriptome response to RPA was also significant in the radar map. Some studies have found that PF can increase serotonin (5-HT) and 5-hydroxyindoleacetic acid in the prefrontal cortex and hippocampus in post-traumatic stress disorder. And, the levels of corticosterone, corticotropin releasing hormone, and adrenocorticotropic hormone in serum were also reversed by PF [28]. This finding suggested that RPA may exert an effect on mental disease by regulating the HPA axis.

## Discussion

At present, numbers of studies have found the therapeutic effect of RPA and its main components on lipid metabolism-related diseases. Such as RPA can reverse the abnormal lipid profiles in serum induced by alcohol in conjunction with a high fat diet [29]. In addition, RPA can reduce the serum TG, malondialdehyde, leptin and TNF-α levels caused by ovariectomies, so as to improve the lipid metabolism disorder and inhibit obesity [30]. These results suggest that RPA can be used in the clinical treatment of steatohepatitis, as well as the obesity caused by the decrease of estrogen levels in women during and after perimenopausal period, relevant mechanism is still unclear.

In this study, we found that RPA played the most significant role on the transcriptome of the liver, especially the glycerolipid metabolism pathway, and the effect may be linked to the metabolism of glucose, lipids, amino acids pathways of the body. The result is consistent with the content of “RPA to liver meridian” in the traditional theory to a certain extent. However, the method established in this study cannot simply correspond to the theory of meridian tropism. Since the liver and brain are the main sites where RPA exerted an effect, which was different from the traditional functional characteristics of RPA of “acting on the liver meridian and spleen meridian”, as there is no “brain meridian” in the theory of TCM. In fact, a new interpretation and supplement of RPA medicinal content were proposed based on this modern research methods. The response intensity of the spleen transcriptome to RPA was not prominent in the whole model, which may be due to the functions of the spleen in modern medicine and those of the “spleen” in TCM theory being quite different.

It still needs to be noted that although organs in TCM theory cannot be completely equated with modern medicine, they have a high degree of consistency in the function of the liver. For example, it is believed that liver has two main functions, namely “controlling conveyance and dispersion” and “storing blood” in TCM. The former is related to digestive function, fluid metabolism and reproductive function, etc. The latter can regulate blood volume and prevent abnormal haemorrhage. In modern medicine, the liver is also an important digestive and metabolic organs with the function of excreting bile, storing glycogen, and participating in the regulation of nutrients synthesis and degradation. In addition, liver participate in the metabolism of water, hormone inactivation, and synthesizing plasma albumin, plasma globulin, and coagulation factors. RPA is generally considered as an herb of liver meridian with a series of effects such as “collect liver Yin, nourishing the liver blood, soften liver body “, etc. since the definition of organs in TCM is more based on the physiological functions.

Previous research showed that there was a negative correlation between mature Brain-derived neurotrophic factor in parietal cortex and in liver which indicated that there is a liver-brain axis in psychiatric disorders [31]. In our results, RPA seemed to have great potential value in the treatment of mental diseases because choline seemed to mediate the crosslink of the liver and brain when analyzing combined with metabolomics, and choline could regulate the production of IL-1β and IL-18 by macrophages and affect the acute and chronic inflammation models [32]. These findings suggested that RPA may influence immune activity by regulating the metabolic processes of choline.

In addition, immune-related activities were involved in transcriptomic differential gene expression analysis in multiple organs, such as the Toll-like receptor signaling pathway (lung and adrenal gland), complement and coagulation cascades (heart and liver), the chemokine signaling pathway (liver and spleen), and hematopoietic cell lineage (liver, spleen, lung and brain), which may be the reason why RPA can effectively treat a variety of infectious diseases due to bacteria, viruses and parasites, such as *Staphylococcus aureus* infection, influenza A and leishmaniasis (Table S5). These conclusions, to a certain extent, also provide an interpretation of the TCM theory that RPA can “clear heat and relieve pain”. The combined analysis suggested that TCM have the advantage of multi-circuit comprehensive therapeutic action, that is, multiple metabolic pathways in the body are simultaneously regulated [33].

There are many potential mechanisms of the pharmacological efficacy of RPA that worth to be discussed. For instance, RPA has an effect of “dispersing stagnated liver ‘qi’ and relieving depression” in TCM theory. Although it was not directly related to depression-related pathways in the results, as we’ve known before, a deficit in GABAergic transmission in neural circuits is causal for depression. Inversely, an enhancement of GABA transmission has antidepressant effects [34], and the genes encode GABA receptors, such as Gabrb1, Gabre and Gabrq, were significantly increased as well as the GABAergic synapse pathway were directly enriched. These unexplored link like this needs to be further explored in the future.

## Conclusions

In this study, transcriptomics and metabolomics were integrated to provide an unexpected and unbiased analysis of the effective profile of in vivo administration of RPA, which is a frequently used natural medicine in TCM. First, the changes in serum metabolites were evaluated, and we found that RPA had certain effects on energy metabolism. By using high-throughput sequencing technology to detect the transcriptome of organs, we found that the liver is the organ with most obvious responses to RPA administration, which is very consistent with the theory that “RPA goes to the liver meridian” in TCM. Combined with serum metabolomics, we further found that RPA plays a role in regulating lipid metabolism by regulating the expression of enzymes in the glycerolipid metabolism pathway and inducing a decrease in downstream lipid metabolites. In addition, RPA also exerted an important influence on brain tissue, and for the first time, we unexpectedly found that RPA is involved in regulating the processes of various substance addiction diseases. To clearly visualize this collaborative regulation pattern, a computational model was designed, which took transcriptomics data as evaluation elements, and the overall function characteristics of RPA were innovatively expressed in a radar map.

This study provided a valuable reference pattern to interpret the theories of TCM, expanded the potential application of RPA, and provided possible targets and directions for further mechanism study.

## Supplementary information

**Additional file 1: Fig. S1** Fuzzification of transcriptome DEGs into 4 terms.

**Additional file 2: Table S1** The number of DEGs and corresponding weight values.

**Additional file 3: Fig. S2** Typical fingerprints of RPA quality control by HPLC.

**Additional file 4: Table S2** Result report of HPLC analysis.

**Additional file 5: Fig. S3** Typical 600 MHz 1H NMR spectra of serum from CON and RPA groups. The dotted region was vertically expanded 32 times in the spectra. TMAO: trimethylamine N-oxide; TG: triglycerides; GPC: glycerophosphocholine; PC: phosphorylcholine; DMG: dimethylglycine; OAG: O-acetylated glycoproteins; NAG: N-acetylated glycoproteins; 3-HB: 3-Hydroxybutytrate.

**Additional file 6: Table S3** Recognition accuracy and time of different dimensionality reduction algorithms combine different classification algorithms.

**Additional file 7: Table S4** Differential metabolites pathway enrichment.

**Additional file 8: Fig. S4 (A)** Enrichment analysis of differential metabolites based on SMPDB. **(B)** Enriched pathways of RPA targets from the TCMSP overlapped with the pathways of liver transcriptomics enrichment caused by RPA. **(C)** Enriched pathways of DEGs overlapped with GSEA based on KEGG. The pathway in the red box was also enriched, according to metabolomics.

**Additional file 9: Table S5** Liver transcriptomics differential genes pathway enrichment.

**Additional file 10: Table S6** Main active ingredients in RPA.

**Additional file 11: Table S7** GSEA results of liver transcriptomic based on KEGG database.

**Additional file 12: Fig. S5** GSEA results showing the pathways that overlap.

**Additional file 13: Table S8** Pathways obtained from the Venn result of liver GSEA and DEG analysis.

**Additional file 14: Fig. S6** Levels of differential lipid metabolites in the serum NMR metabolic spectra, p ˂ 0.05.

**Additional file 15: Fig. S7** Neuroactive ligand-receptor interaction pathway (mmu04080, in KEGG); the red boxes are genes upregulated by RPA, and the blue boxes are genes downregulated by RPA.

**Additional file 16: Fig. S8** PPI analysis network of substance addiction-related disease pathways of brain transcriptomics.

**Additional file 17: Table S9** The first principal component contribution rate of each dimension.

## Data Availability

The datasets generated for this study can be found in the Sequence Read Archive (SRA) database with accession number PRJNA587068 from the National Center for Biotechnology Information, USA.

## Abbreviations

RPA: Radix Paeoniae Alba
CON: Control
TCM: Traditional Chinese Medicine
NMR: Proton nuclear magnetic resonance spectrometer
GO: Gene ontology
KEGG: Kyoto Encyclopedia of Genes and Genomes
PF: Paeoniflorin
HPLC: High-performance liquid chromatography
DEGs: Differentially expressed genes
OPLS-DA: Orthogonal partial least squares discrimination analysis
VIP: Variable importance in the projection value
TCMSP: Traditional Chinese Medicine Systems Pharmacology Database and Analysis Platform
PCA: Principal component analysis
SVM: Support Vector Machine
GPC: Glycerophosphocholine
PC: Phosphorylcholine
NAG: *N*-Acetylated protein
TG: Triglycerides
GSEA: Gene Set Enrichment Analysis
GEO: Gene expression omnibus
