# Supplementary figures and images for: Transcriptomics- and metabolomics-based integration analyses revealed the potential pharmacological effects and functional pattern of in vivo Radix Paeoniae Alba administration
Source: Chin Med. 2020 May 24;15:52. doi: 10.1186/s13020-020-00330-0 (PMC7245909; doi:10.1186/s13020-020-00330-0)

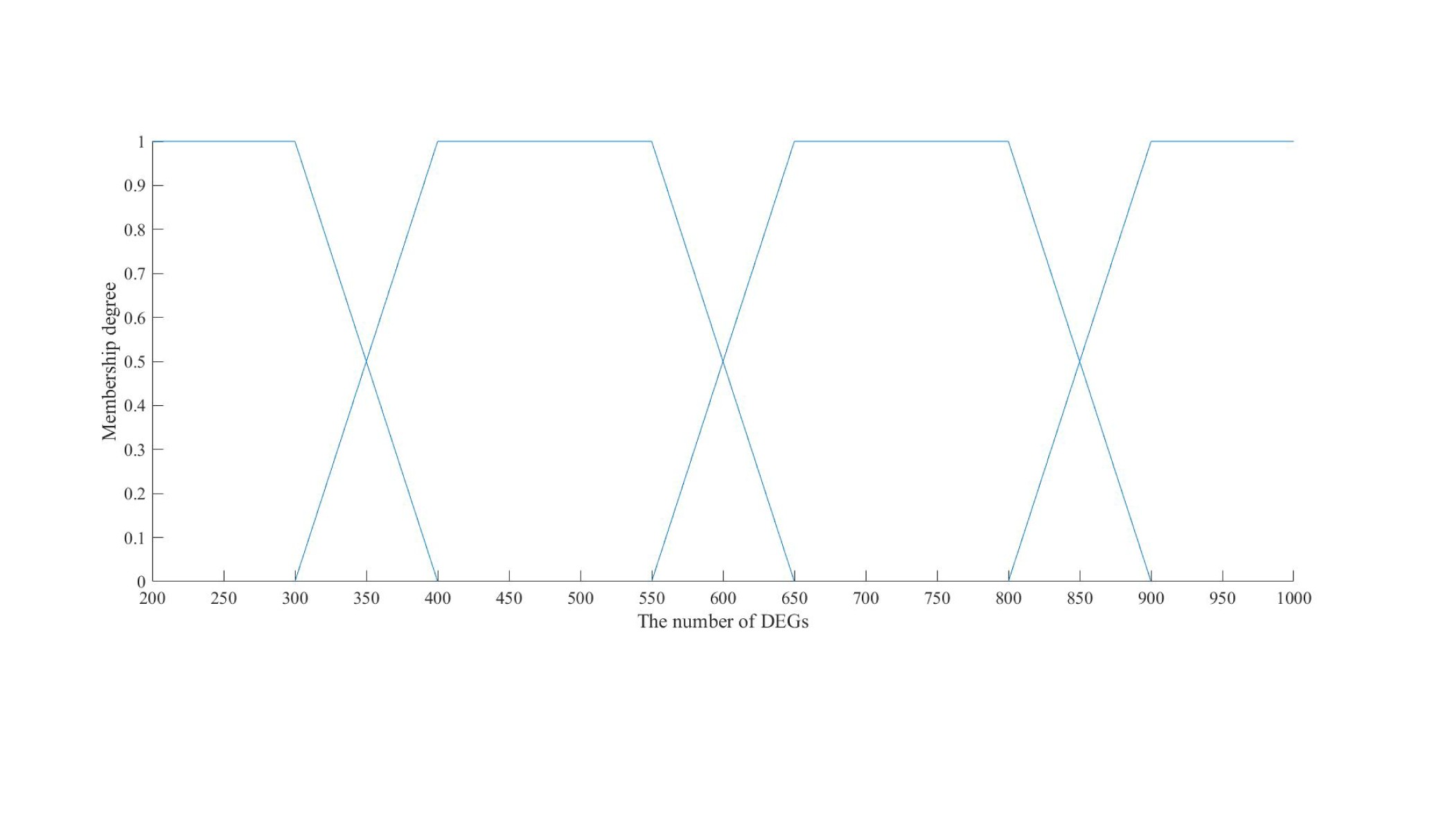

Supplement: Supplementary file 1 — Additional file 1: Fig. S1 Fuzzification of transcriptome DEGs into 4 terms. [file 13020_2020_330_MOESM1_ESM.tif]

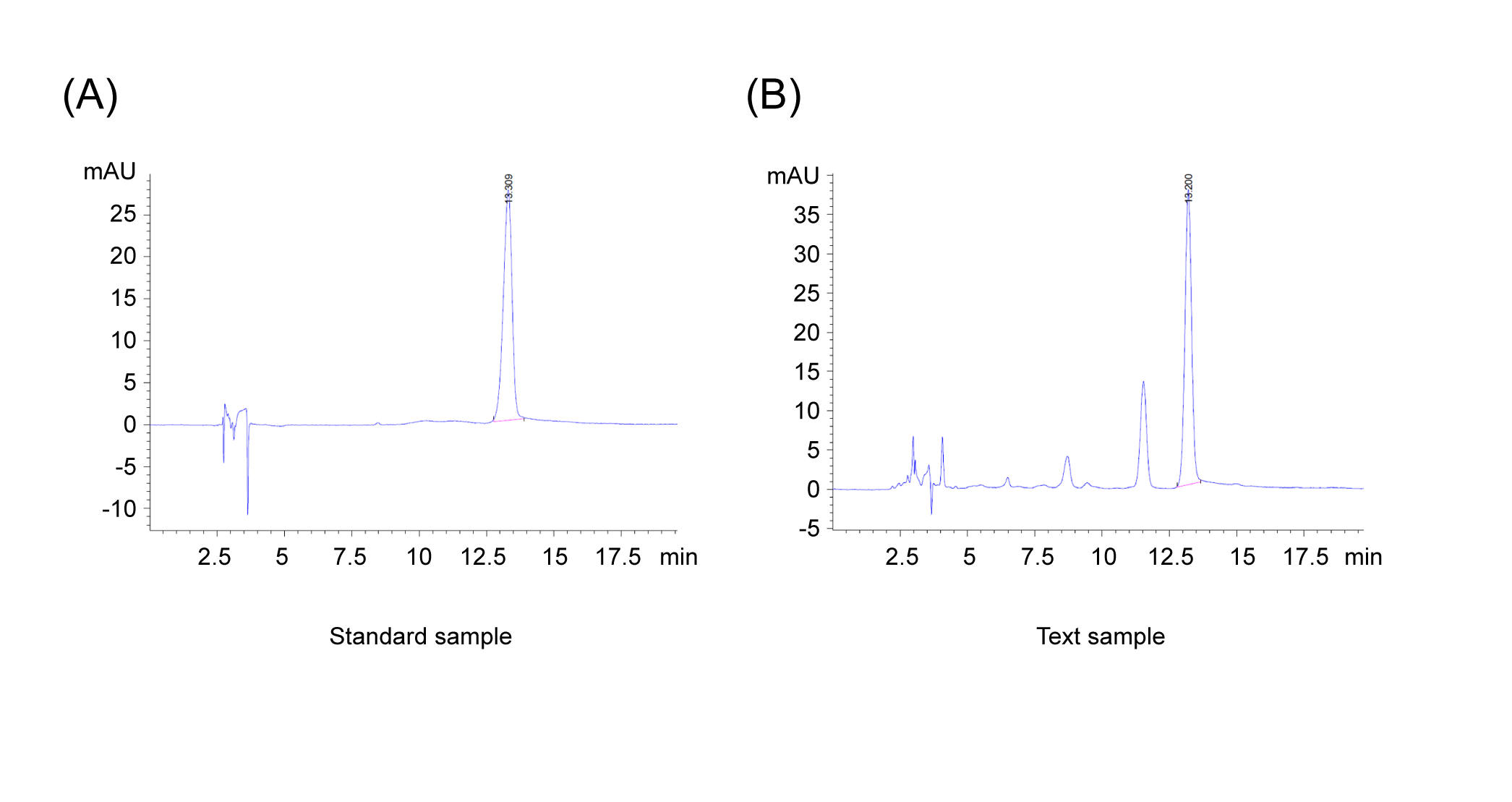

Supplement: Supplementary file 3 — Additional file 3: Fig. S2 Typical fingerprints of RPA quality control by HPLC. [file 13020_2020_330_MOESM3_ESM.tif]

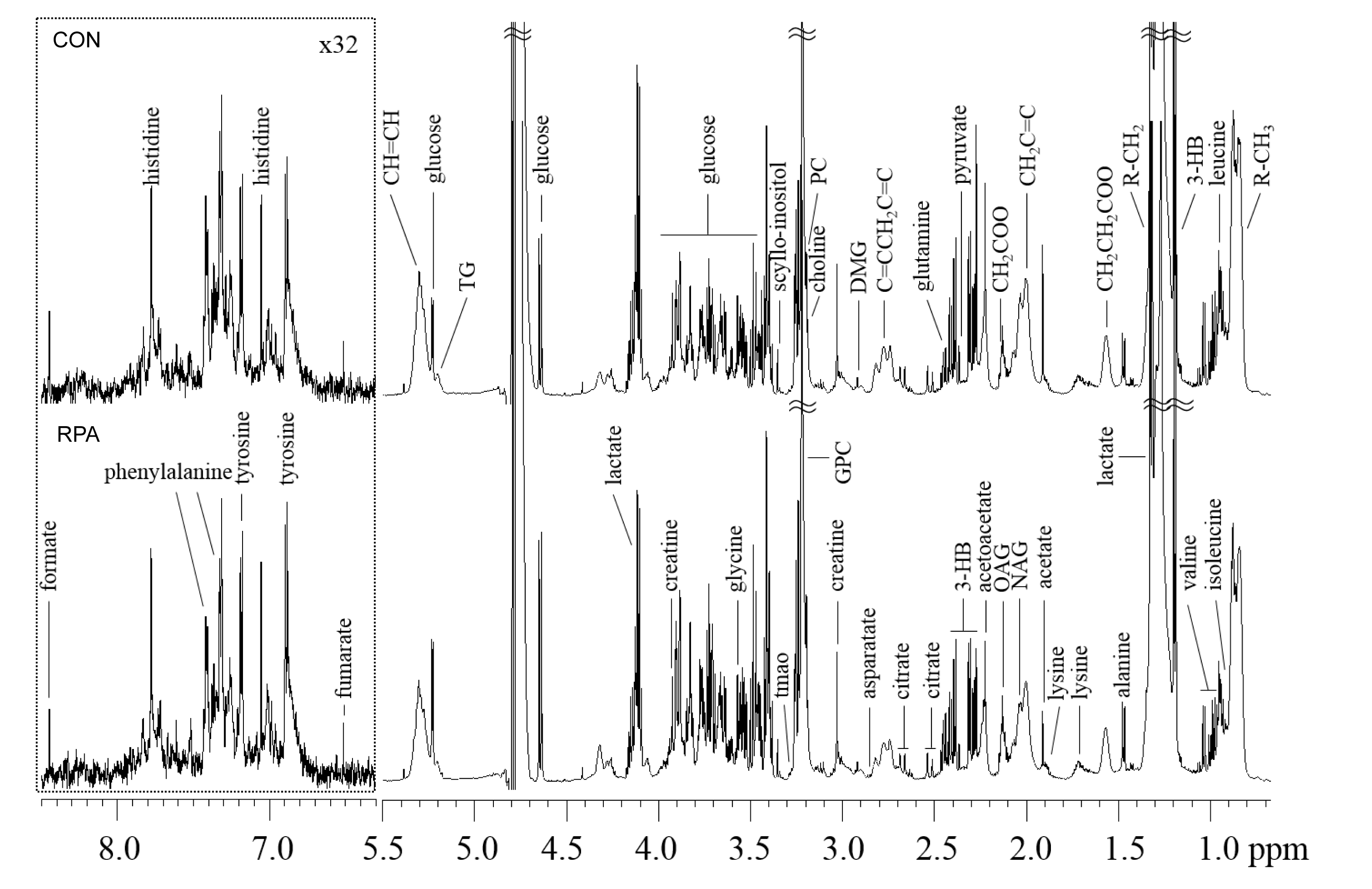

Supplement: Supplementary file 5 — Additional file 5: Fig. S3 Typical 600 MHz 1H NMR spectra of serum from CON and RPA groups. The dotted region was vertically expanded 32 times in the spectra. TMAO: trimethylamine N-oxide; TG: triglycerides; GPC: glycerophosphocholine; PC: phosphorylcholine; DMG: dimethylglycine; OAG: O-acetylated glycoproteins; NAG: N-acetylated glycoproteins; 3-HB: 3-Hydroxybutytrate. [file 13020_2020_330_MOESM5_ESM.tif]

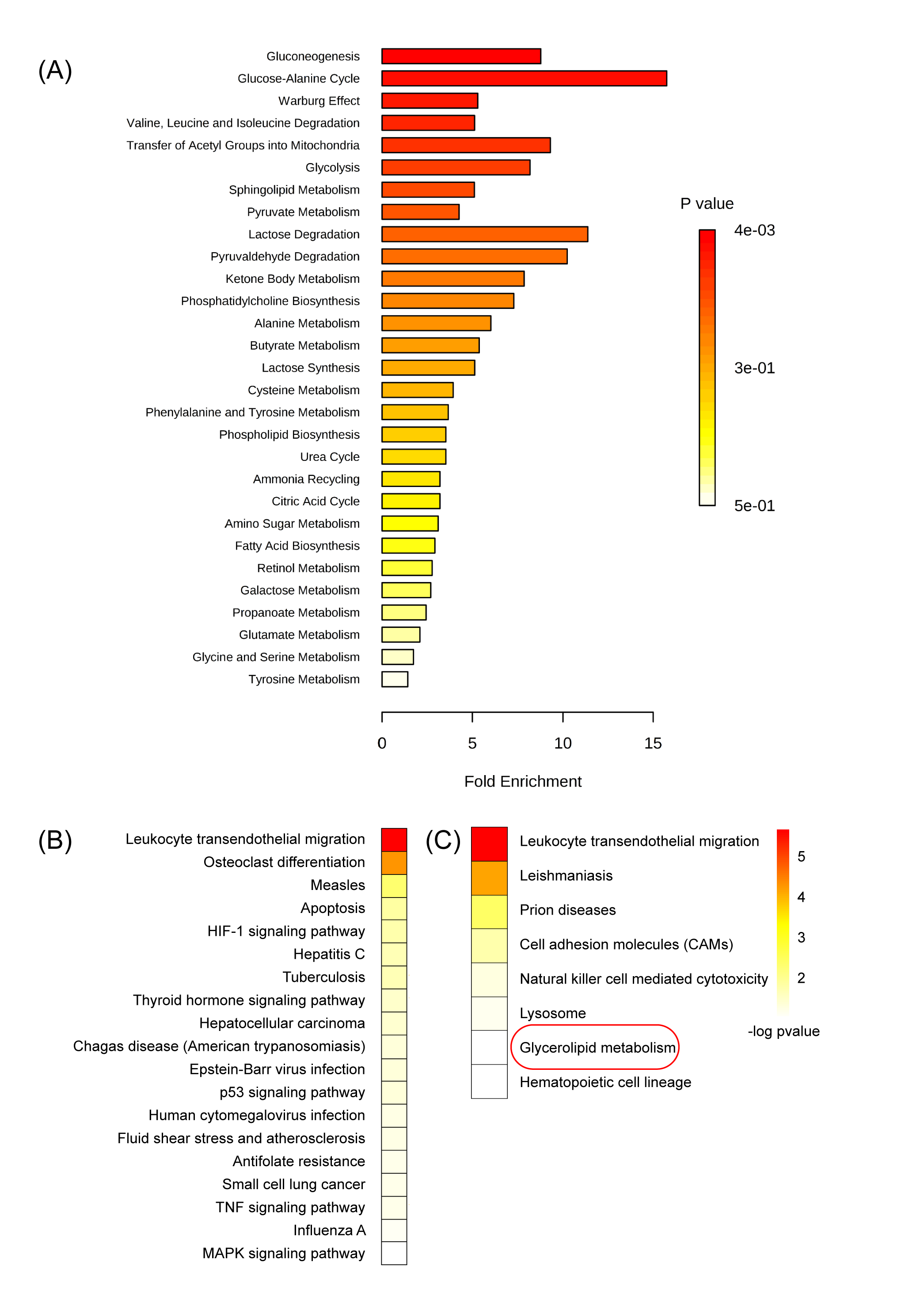

Supplement: Supplementary file 8 — Additional file 8: Fig. S4 (A) Enrichment analysis of differential metabolites based on SMPDB. (B) Enriched pathways of RPA targets from the TCMSP overlapped with the pathways of liver transcriptomics enrichment caused by RPA. (C) Enriched pathways of DEGs overlapped with GSEA based on KEGG. The pathway in the red box was also enriched, according to metabolomics. [file 13020_2020_330_MOESM8_ESM.tif]

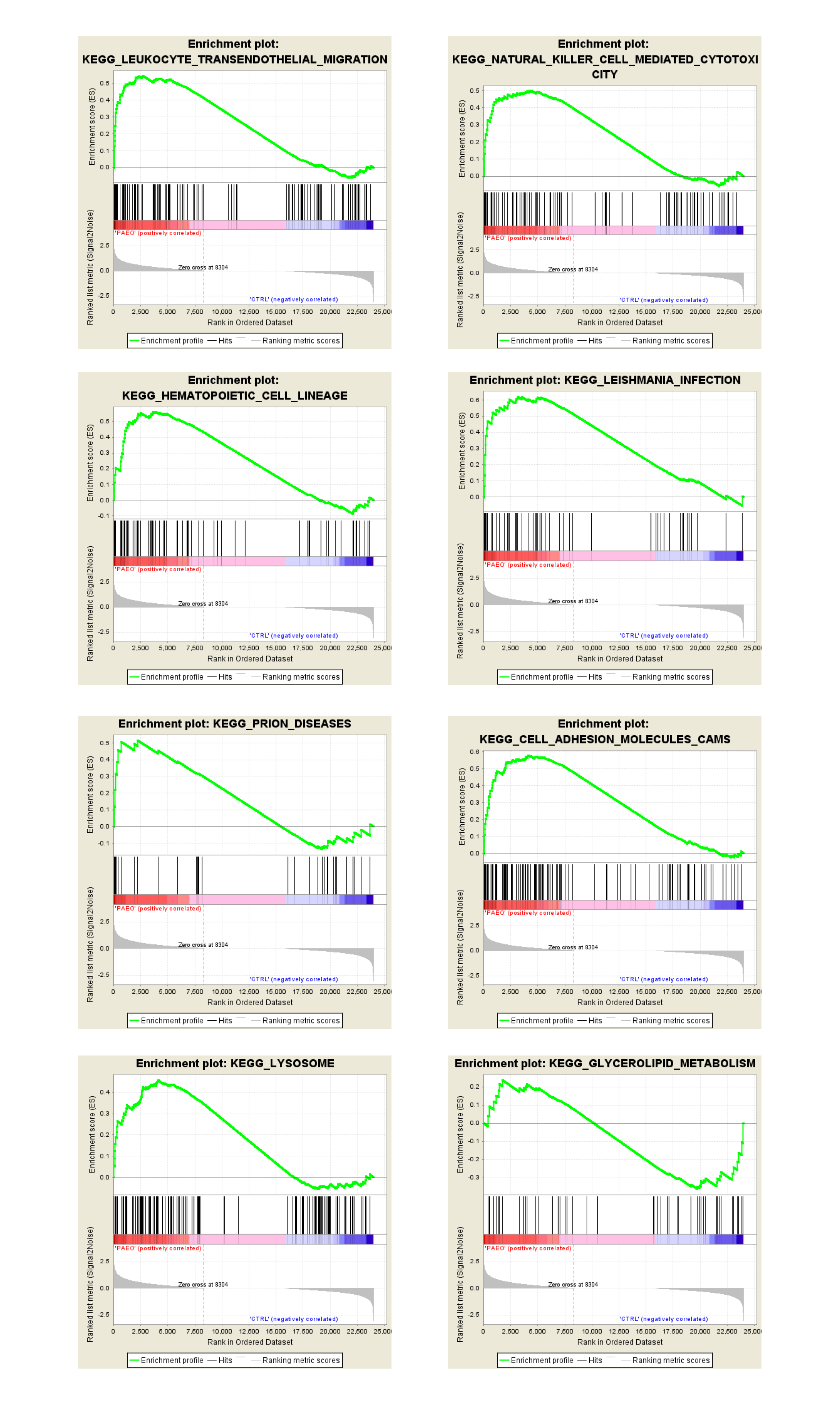

Supplement: Supplementary file 12 — Additional file 12: Fig. S5 GSEA results showing the pathways that overlap. [file 13020_2020_330_MOESM12_ESM.tif]

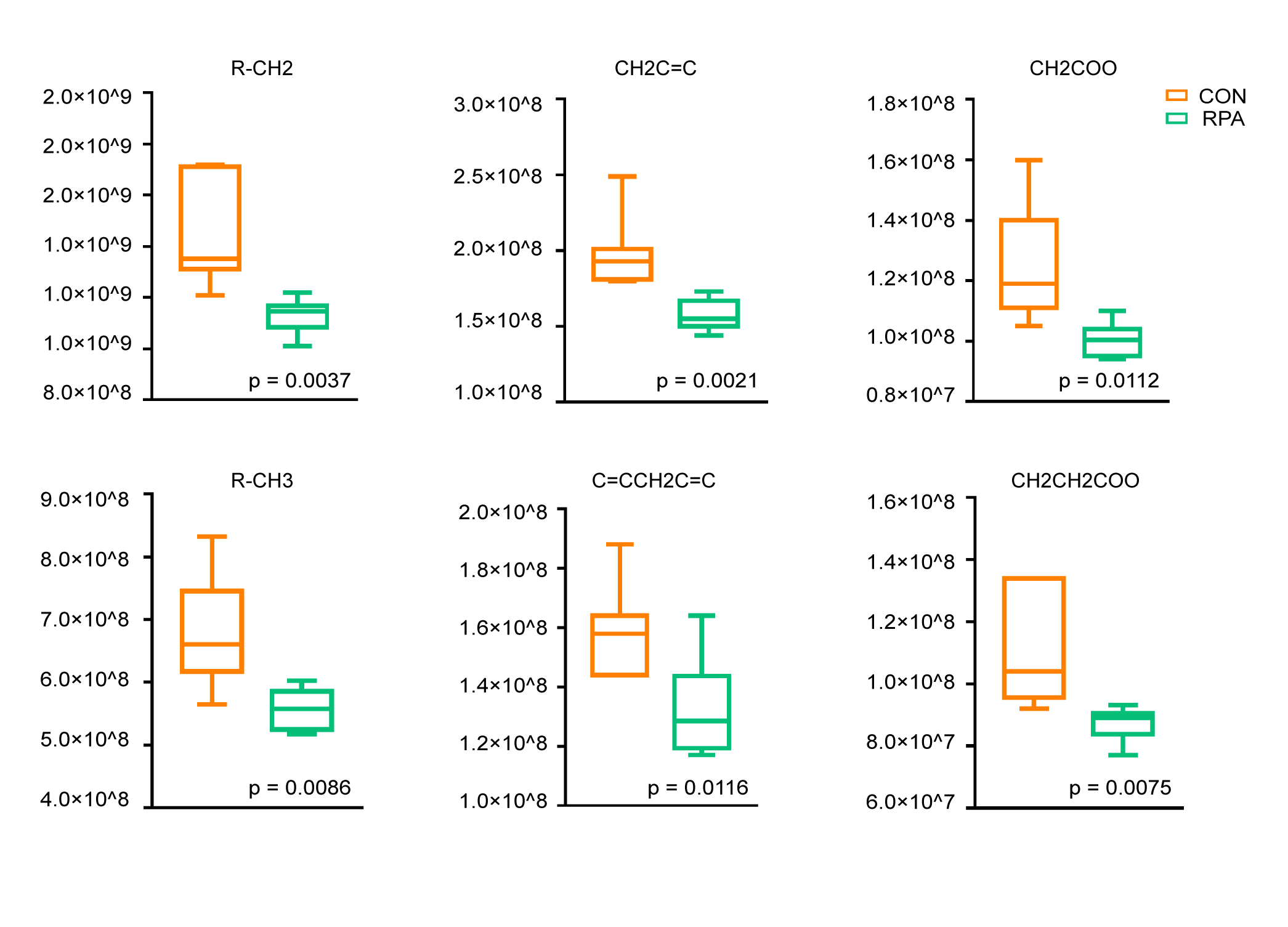

Supplement: Supplementary file 14 — Additional file 14: Fig. S6 Levels of differential lipid metabolites in the serum NMR metabolic spectra, p ˂ 0.05. [file 13020_2020_330_MOESM14_ESM.tif]

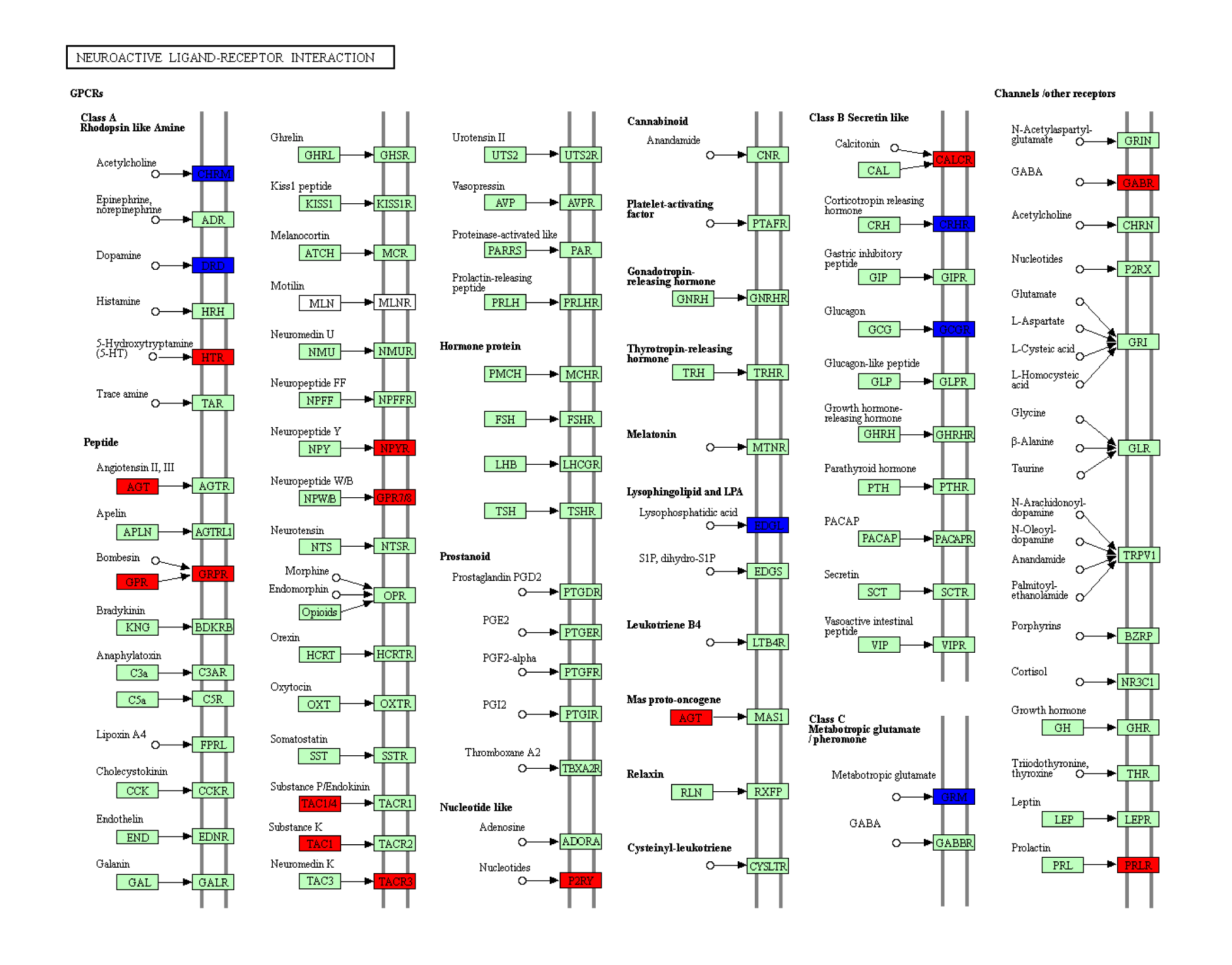

Supplement: Supplementary file 15 — Additional file 15: Fig. S7 Neuroactive ligand-receptor interaction pathway (mmu04080, in KEGG); the red boxes are genes upregulated by RPA, and the blue boxes are genes downregulated by RPA. [file 13020_2020_330_MOESM15_ESM.tif]

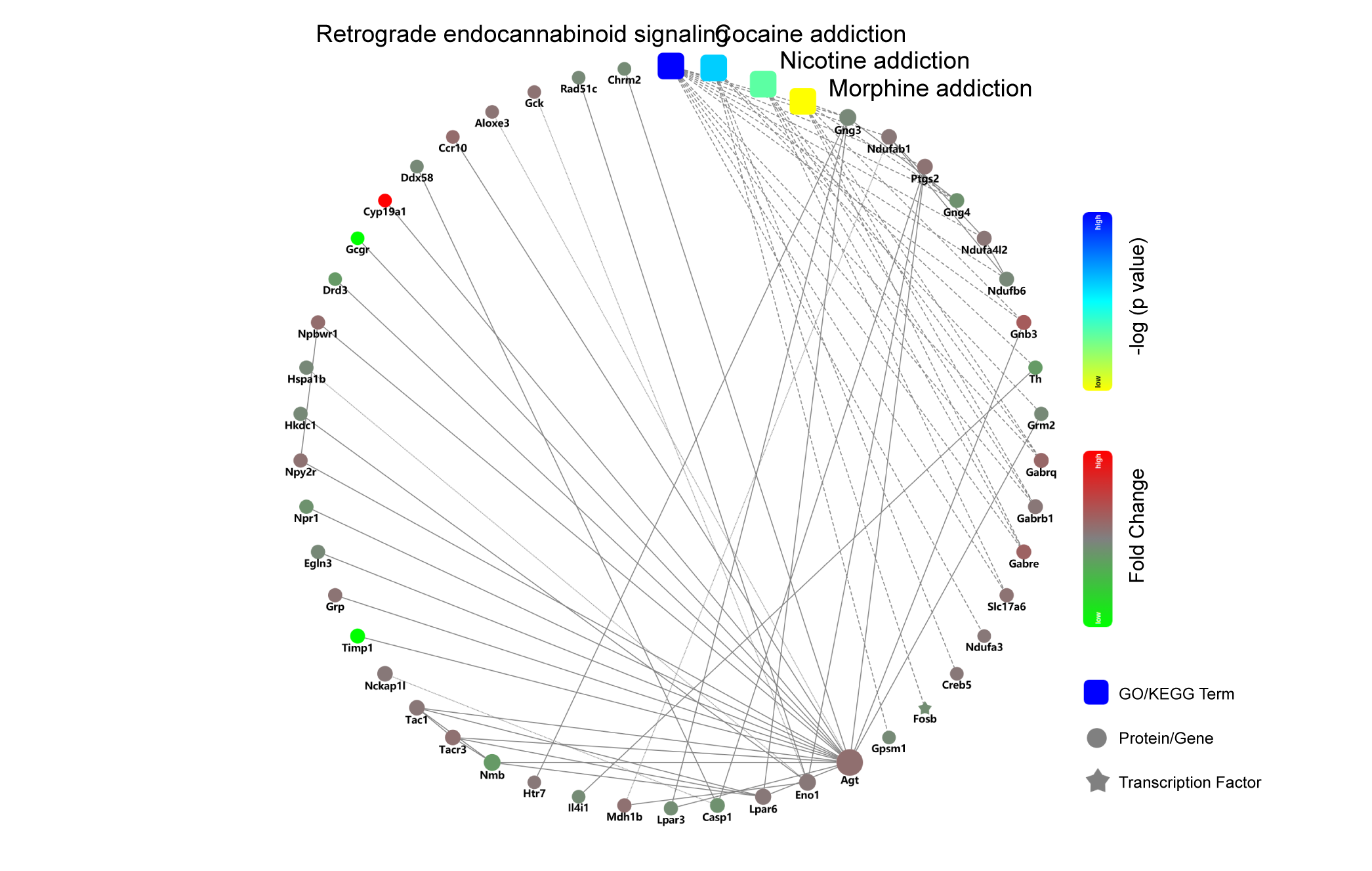

Supplement: Supplementary file 16 — Additional file 16: Fig. S8 PPI analysis network of substance addiction-related disease pathways of brain transcriptomics. [file 13020_2020_330_MOESM16_ESM.tif]
