# Supplementary material for: Transcriptomics- and metabolomics-based integration analyses revealed the potential pharmacological effects and functional pattern of in vivo Radix Paeoniae Alba administration
Source: Chin Med. 2020 May 24;15:52. doi: 10.1186/s13020-020-00330-0 (PMC7245909; doi:10.1186/s13020-020-00330-0)
Supplement: Supplementary file 2 — Additional file 2: Table S1 The number of DEGs and corresponding weight values. [file 13020_2020_330_MOESM2_ESM.docx]

**Additional file: Table S1** The number of DEGs and corresponding weight values

| **Group** | **Adrenal** | **Brain** | **Heart** | **Kidney** | **Liver** | **Lung** | **Spleen** |
| --- | --- | --- | --- | --- | --- | --- | --- |
| DEGs | 753 | 625 | 394 | 457 | 920 | 697 | 423 |
| Weight values | 0.7 | 0.7 | 0.5 | 0.5 | 0.9 | 0.7 | 0.5 |
