# Supplementary material for: Transcriptomics- and metabolomics-based integration analyses revealed the potential pharmacological effects and functional pattern of in vivo Radix Paeoniae Alba administration
Source: Chin Med. 2020 May 24;15:52. doi: 10.1186/s13020-020-00330-0 (PMC7245909; doi:10.1186/s13020-020-00330-0)
Supplement: Supplementary file 4 — Additional file 4: Table S2 Result report of HPLC analysis. [file 13020_2020_330_MOESM4_ESM.docx]

**Additional file: Table S2** Result report of HPLC analysis

| **Sample** | **Ret Time [min]** | **Width [min]** | **Area [mAU*s]** | **Height** |
| --- | --- | --- | --- | --- |
| Text 1-1 | 13.200 | 0.2689 | 653.28961 | 37.55521 |
| Text 1-2 | 13.343 | 0.2677 | 653.03442 | 37.39402 |
| Text 2-1 | 13.270 | 0.2726 | 658.66046 | 37.56524 |
| Text 2-2 | 13.377 | 0.2743 | 659.22516 | 37.27192 |
| Standard | 13.309 | 0.3480 | 616.14014 | 27.43537 |
