# Supplementary material for: Transcriptomics- and metabolomics-based integration analyses revealed the potential pharmacological effects and functional pattern of in vivo Radix Paeoniae Alba administration
Source: Chin Med. 2020 May 24;15:52. doi: 10.1186/s13020-020-00330-0 (PMC7245909; doi:10.1186/s13020-020-00330-0)
Supplement: Supplementary file 6 — Additional file 6: Table S3 Recognition accuracy and time of different dimensionality reduction algorithms combine different classification algorithms. [file 13020_2020_330_MOESM6_ESM.docx]

**Additional file: Table S3** Recognition accuracy and time of different dimensionality reduction algorithms combine different classification algorithms

| **Model** | **Recognition rate（%）** | **Time (ms)** |
| --- | --- | --- |
| PCA(3 dims) + NB | 80 | 17.6118 |
| PCA(3 dims) + RF | 80 | 304.2058 |
| PCA(3 dims) + BPNN | 80 | 58.9392 |
| PCA(3 dims) + KNN | 100 | 47.66 |
| PCA(3 dims) + SVM | 100 | 0.6066 |
| T-SNE(3 dims) + NB | 100 | 15.7366 |
| T-SNE (3 dims) + RF | 100 | 293.3104 |
| T-SNE (3 dims) + BPNN | 40 | 57.272 |
| T-SNE (3 dims) + KNN | 80 | 48.4754 |
| T-SNE (3 dims) + SVM | 80 | 0.5298 |
| LAP(3 dims) + NB | 80 | 18.024 |
| LAP (3 dims) + RF | 80 | 299.2726 |
| LAP (3 dims) + BPNN | 60 | 53.2376 |
| LAP (3 dims) + KNN | 60 | 48.9032 |
| LAP (3 dims) + SVM | 80 | 0.4702 |
| ISO(3 dims) + NB | 80 | 16.2066 |
| ISO (3 dims) + RF | 80 | 331.7222 |
| ISO (3 dims) + BPNN | 60 | 48.2318 |
| ISO (3 dims) + KNN | 100 | 52.9288 |
| ISO (3 dims) + SVM | 100 | 0.4452 |
| LLE(3 dims) + NB | 100 | 15.7744 |
| LLE (3 dims) + RF | 60 | 293.3298 |
| LLE (3 dims) + BPNN | 40 | 49.5046 |
| LLE (3 dims) + KNN | 80 | 48.0818 |
| LLE (3 dims) + SVM | 80 | 0.6458 |
| WT(3 dims) + NB | 80 | 24.6562 |
| WT (3 dims) + RF | 60 | 339.2038 |
| WT (3 dims) + BPNN | 40 | 63.4066 |
| WT (3 dims) + KNN | 100 | 51.375 |
| WT (3 dims) + SVM | 80 | 1.3986 |

Abbreviations: PCA, Principal Component Analysis; T-SNE, T-distributed Stochastic Neighbor Embedding; LAP, Laplacian Eigenmaps; ISO, Isometric Mapping; LLE, Locally Linear Embedding; WT, Wavelet Transform; NB, Naive Bayesian; RF, Random Forest; BPNN, Back-Propagation Neural Network; KNN, k-Nearest Neighbor; SVM, Support Vector Machine.
