# Supplementary material for: Transcriptomics- and metabolomics-based integration analyses revealed the potential pharmacological effects and functional pattern of in vivo Radix Paeoniae Alba administration
Source: Chin Med. 2020 May 24;15:52. doi: 10.1186/s13020-020-00330-0 (PMC7245909; doi:10.1186/s13020-020-00330-0)
Supplement: Supplementary file 7 — Additional file 7: Table S4 Differential metabolites pathway enrichment. [file 13020_2020_330_MOESM7_ESM.docx]

**Additional file: Table S4** Differential metabolites pathway enrichment

| **Pathway name** | **Total** | **Expected** | **Raw p** |
| --- | --- | --- | --- |
| Valine, leucine and isoleucine biosynthesis | 11 | 0.077629 | 4.06E-05 |
| Valine, leucine and isoleucine degradation | 38 | 0.26817 | 0.001877 |
| Glycerolipid metabolism | 18 | 0.12703 | 0.00646 |
| Butanoate metabolism | 22 | 0.15526 | 0.009607 |
| Pyruvate metabolism | 23 | 0.16231 | 0.010483 |
| Glycolysis or Gluconeogenesis | 26 | 0.18349 | 0.013314 |
| Glycerophospholipid metabolism | 30 | 0.21171 | 0.017553 |
| Synthesis and degradation of ketone bodies | 5 | 0.035286 | 0.03484 |
| Aminoacyl-tRNA biosynthesis | 69 | 0.48694 | 0.081683 |
| Pantothenate and CoA biosynthesis | 15 | 0.10586 | 0.10126 |
| Starch and sucrose metabolism | 19 | 0.13409 | 0.12666 |
| Citrate cycle (TCA cycle) | 20 | 0.14114 | 0.1329 |
| Alanine, aspartate and glutamate metabolism | 24 | 0.16937 | 0.15749 |
| Galactose metabolism | 26 | 0.18349 | 0.16955 |
| Cysteine and methionine metabolism | 27 | 0.19054 | 0.17552 |
| Glycine, serine and threonine metabolism | 31 | 0.21877 | 0.19902 |
| Fatty acid metabolism | 39 | 0.27523 | 0.24421 |
| Tyrosine metabolism | 44 | 0.31052 | 0.27128 |
