# Supplementary material for: Transcriptomics- and metabolomics-based integration analyses revealed the potential pharmacological effects and functional pattern of in vivo Radix Paeoniae Alba administration
Source: Chin Med. 2020 May 24;15:52. doi: 10.1186/s13020-020-00330-0 (PMC7245909; doi:10.1186/s13020-020-00330-0)
Supplement: Supplementary file 9 — Additional file 9: Table S5 Liver transcriptomics differential genes pathway enrichment. [file 13020_2020_330_MOESM9_ESM.docx]

**Additional file: Table S5** Liver transcriptomics differential genes pathway enrichment

| **Pathway Name** | **Pvalue** | **Subunit Category** | **Class** |
| --- | --- | --- | --- |
| Starch and sucrose metabolism | 9.40E-03 | Carbohydrate metabolism | Metabolism |
| Fructose and mannose metabolism | 4.97E-02 |  |  |
| Glycerophospholipid metabolism | 1.53E-02 | Lipid metabolism |  |
| Glycerolipid metabolism | 3.44E-02 |  |  |
| Sphingolipid metabolism | 4.18E-02 |  |  |
| Pyrimidine metabolism | 2.77E-02 | Nucleotide metabolism |  |
| Purine metabolism | 4.59E-02 |  |  |
| HIF-1 signaling pathway | 9.13E-03 | Signal tranduction | Environmental Information Processing |
| AMPK signaling pathway | 1.20E-02 |  |  |
| TNF signaling pathway | 3.18E-02 |  |  |
| MAPK signaling pathway | 4.77E-02 |  |  |
| Cell adhesion molecules (CAMs) | 7.75E-03 | Signaling molecules and interaction |  |
| Phagosome | 1.09E-04 | Transport and catabolism | Cellular Processes |
| Lysosome | 2.66E-02 |  |  |
| Apoptosis | 8.20E-03 | Cell growth and death |  |
| p53 signaling pathway | 2.29E-02 |  |  |
| Tight junction | 2.79E-03 | Cellular community |  |
| Adherens junction | 2.45E-02 |  |  |
| Leukocyte transendothelial migration | 2.83E-06 | Immune system | Organismal Systems |
| Fc gamma R-mediated phagocytosis | 6.58E-04 |  |  |
| Complement and coagulation cascades | 2.59E-03 |  |  |
| Natural killer cell mediated cytotoxicity | 1.96E-02 |  |  |
| Chemokine signaling pathway | 2.96E-02 |  |  |
| Hematopoietic cell lineage | 3.60E-02 |  |  |
| PPAR signaling pathway | 5.38E-04 | Endocrine system |  |
| Thyroid hormone signaling pathway | 1.66E-02 |  |  |
| Glucagon signaling pathway | 2.07E-02 |  |  |
| Osteoclast differentiation | 4.78E-05 | Development |  |
| Circadian rhythm | 3.04E-02 | Environmental adaptation |  |
| Pathways in cancer | 8.34E-03 | Cancers: Overview | Human Diseases |
| Bladder cancer | 2.29E-02 | Cancers: Specific types |  |
| Small cell lung cancer | 3.05E-02 |  |  |
| Non-small cell lung cancer | 4.78E-02 |  |  |
| Prion diseases | 2.04E-03 | Neurodegenerative diseases |  |
| Morphine addiction | 3.05E-02 | Substance dependece |  |
| Staphylococcus aureus infection | 4.20E-04 | Infectious diseases: Bacterial |  |
| Tuberculosis | 1.20E-02 |  |  |
| Measles | 3.06E-03 | Infectious diseases: Viral |  |
| Hepatitis C | 1.18E-02 |  |  |
| Epstein-Barr virus infection | 2.21E-02 |  |  |
| Influenza A | 3.70E-02 |  |  |
| Leishmaniasis | 6.16E-05 |  |  |
| Chagas disease (American trypanosomiasis) | 2.19E-02 |  |  |
| Neomycin, kanamycin and gentamicin biosynthesis | 1.47E-02 | Other and unknow | |
| Phospholipase D signaling pathway | 1.62E-02 |  |  |
| Cellular senescence | 1.70E-02 |  |  |
| Hepatocellular carcinoma | 1.95E-02 |  |  |
| Human cytomegalovirus infection | 2.78E-02 |  |  |
| Fluid shear stress and atherosclerosis | 2.81E-02 |  |  |
| Antifolate resistance | 3.04E-02 |  |  |
| Cholesterol metabolism | 4.51E-02 |  |  |
