# Supplementary material for: Transcriptomics- and metabolomics-based integration analyses revealed the potential pharmacological effects and functional pattern of in vivo Radix Paeoniae Alba administration
Source: Chin Med. 2020 May 24;15:52. doi: 10.1186/s13020-020-00330-0 (PMC7245909; doi:10.1186/s13020-020-00330-0)
Supplement: Supplementary file 10 — Additional file 10: Table S6 Main active ingredients in RPA. [file 13020_2020_330_MOESM10_ESM.docx]

**Additional file: Table S6** Main active ingredients in RPA

| **Mol ID** | **Molecule Name** | **MW** | **OB (%)** | **DL** |
| --- | --- | --- | --- | --- |
| MOL001910 | 11alpha,12alpha-epoxy-3beta-23-dihydroxy-30-norolean-20-en-28,12beta-olide | 470.71 | 64.77 | 0.38 |
| MOL001918 | paeoniflorgenone | 318.35 | 87.59 | 0.37 |
| MOL001919 | (3S,5R,8R,9R,10S,14S)-3,17-dihydroxy-4,4,8,10,14-pentamethyl-2,3,5,6,7,9-hexahydro-1H-cyclopenta[a]phenanthrene-15,16-dione | 358.52 | 43.56 | 0.53 |
| MOL001921 | Lactiflorin | 462.49 | 49.12 | 0.8 |
| MOL001924 | paeoniflorin | 480.51 | 53.87 | 0.79 |
| MOL001925 | paeoniflorin | 318.35 | 68.18 | 0.4 |
| MOL001928 | albiflorin | 318.35 | 66.64 | 0.33 |
| MOL001930 | benzoyl paeoniflorin | 584.62 | 31.27 | 0.75 |
| MOL000211 | Mairin | 456.78 | 55.38 | 0.78 |
| MOL000358 | beta-sitosterol | 414.79 | 36.91 | 0.75 |
| MOL000359 | sitosterol | 414.79 | 36.91 | 0.75 |
| MOL000422 | kaempferol | 286.25 | 41.88 | 0.24 |
| MOL000492 | (+)-catechin | 290.29 | 54.83 | 0.24 |
