# Supplementary material for: Transcriptomics- and metabolomics-based integration analyses revealed the potential pharmacological effects and functional pattern of in vivo Radix Paeoniae Alba administration
Source: Chin Med. 2020 May 24;15:52. doi: 10.1186/s13020-020-00330-0 (PMC7245909; doi:10.1186/s13020-020-00330-0)
Supplement: Supplementary file 11 — Additional file 11: Table S7 GSEA results of liver transcriptomic based on KEGG database. [file 13020_2020_330_MOESM11_ESM.docx]

**Additional file: Table S7** GSEA results of liver transcriptomic based on KEGG database

| **NAME** | **NES** | **p value** |
| --- | --- | --- |
| KEGG_REGULATION_OF_AUTOPHAGY | -1.30688 | 0 |
| KEGG_VASOPRESSIN_REGULATED_WATER_REABSORPTION | -1.27633 | 0 |
| KEGG_PROSTATE_CANCER | -1.24438 | 0 |
| KEGG_GLYCEROLIPID_METABOLISM | -1.22324 | 0 |
| KEGG_LEUKOCYTE_TRANSENDOTHELIAL_MIGRATION | 1.515153 | 0 |
| KEGG_OTHER_GLYCAN_DEGRADATION | 1.492252 | 0 |
| KEGG_VIRAL_MYOCARDITIS | 1.491299 | 0 |
| KEGG_HISTIDINE_METABOLISM | 1.480952 | 0 |
| KEGG_GLYCOSAMINOGLYCAN_DEGRADATION | 1.452845 | 0 |
| KEGG_LYSOSOME | 1.440249 | 0 |
| KEGG_LEISHMANIA_INFECTION | 1.438316 | 0 |
| KEGG_CELL_ADHESION_MOLECULES_CAMS | 1.43727 | 0 |
| KEGG_GRAFT_VERSUS_HOST_DISEASE | 1.398316 | 0 |
| KEGG_HEMATOPOIETIC_CELL_LINEAGE | 1.379812 | 0 |
| KEGG_BASAL_CELL_CARCINOMA | 1.319819 | 0 |
| KEGG_NATURAL_KILLER_CELL_MEDIATED_CYTOTOXICITY | 1.294448 | 0 |
| KEGG_PRION_DISEASES | 1.281151 | 0 |
| KEGG_HEDGEHOG_SIGNALING_PATHWAY | 1.27123 | 0 |
| KEGG_ARACHIDONIC_ACID_METABOLISM | 1.26001 | 0 |
| KEGG_ETHER_LIPID_METABOLISM | 1.253277 | 0 |
| KEGG_TYPE_I_DIABETES_MELLITUS | 1.201728 | 0 |
