# Supplementary material for: Transcriptomics- and metabolomics-based integration analyses revealed the potential pharmacological effects and functional pattern of in vivo Radix Paeoniae Alba administration
Source: Chin Med. 2020 May 24;15:52. doi: 10.1186/s13020-020-00330-0 (PMC7245909; doi:10.1186/s13020-020-00330-0)
Supplement: Supplementary file 13 — Additional file 13: Table S8 Pathways obtained from the Venn result of liver GSEA and DEG analysis. [file 13020_2020_330_MOESM13_ESM.docx]

**Additional file: Table S8** Pathways obtained from the Venn result of liver GSEA and DEG analysis

| **Pathway Name** | **Category** |
| --- | --- |
| Leukocyte transendothelial migration | Immune system |
| Natural killer cell mediated cytotoxicity |  |
| Hematopoietic cell lineage |  |
| Leishmaniasis | Infectious diseases : Parasitic |
| Prion diseases | Neurodegenerative diseases |
| Cell adhesion molecules (CAMs) | Signaling molecules and interaction |
| Lysosome | Transport and catabolism |
| Glycerolipid metabolism | Lipid metabolism |
