# Supplementary material for: Transcriptomics- and metabolomics-based integration analyses revealed the potential pharmacological effects and functional pattern of in vivo Radix Paeoniae Alba administration
Source: Chin Med. 2020 May 24;15:52. doi: 10.1186/s13020-020-00330-0 (PMC7245909; doi:10.1186/s13020-020-00330-0)
Supplement: Supplementary file 17 — Additional file 17: Table S9 The first principal component contribution rate of each dimension. [file 13020_2020_330_MOESM17_ESM.docx]

**Additional file: Table S9** The first principal component contribution rate of each dimension

| **Dim** | **Adrenal** | **Brain** | **Heart** | **Kidney** | **Liver** | **Lung** | **Spleen** |
| --- | --- | --- | --- | --- | --- | --- | --- |
| PC01 | 70.48% | 91.20% | 95.11% | 86.97% | 86.85% | 91.43% | 81.51% |
